# Innate immune memory through TLR2 and NOD2 contributes to the control of *Leptospira interrogans* infection

**DOI:** 10.1371/journal.ppat.1007811

**Published:** 2019-05-20

**Authors:** Ignacio Santecchia, Frédérique Vernel-Pauillac, Orhan Rasid, Jessica Quintin, Maria Gomes-Solecki, Ivo G. Boneca, Catherine Werts

**Affiliations:** 1 Unité Biologie et Génétique de la Paroi Bactérienne, Institut Pasteur, Groupe Avenir, INSERM, Paris, France; 2 Université Paris Descartes, Sorbonne Paris Cité, Paris, France; 3 Chromatine et Infection G5, Institut Pasteur, Paris, France; 4 Immunologie des infections fongiques G5, Institut Pasteur, Paris, France; 5 University of Tennessee Health Science Center, Department of Microbiology, Immunology and Biochemistry, Memphis, Tennessee, United States of America; University of Toronto, CANADA

## Abstract

*Leptospira interrogans* are pathogenic spirochetes responsible for leptospirosis, a worldwide reemerging zoonosis. Many *Leptospira* serovars have been described, and prophylaxis using inactivated bacteria provides only short-term serovar-specific protection. Therefore, alternative approaches to limit severe leptospirosis in humans and morbidity in cattle would be welcome. Innate immune cells, including macrophages, play a key role in fighting infection and pathogen clearance. Recently, it has been shown that functional reprograming of innate immune cells through the activation of pattern recognition receptors leads to enhanced nonspecific antimicrobial responses upon a subsequent microbial encounter. This mechanism is known as trained immunity or innate immune memory. We have previously shown that oral treatment with *Lactobacillus plantarum* confers a beneficial effect against acute leptospirosis. Here, using a macrophage depletion protocol and live imaging in mice, we established the role of peritoneal macrophages in limiting the initial dissemination of leptospires. We further showed that intraperitoneal priming of mice with CL429, a TLR2 and NOD2 agonist known to mimic the modulatory effect of *Lactobacillus*, alleviated acute leptospiral infection. The CL429 treatment was characterized as a training effect since i.) it was linked to peritoneal macrophages that produced *ex vivo* more pro-inflammatory cytokines and chemokines against 3 different pathogenic serovars of *Leptospira*, independently of the presence of B and T cells, ii.) it had systemic effects on splenic cells and bone marrow derived macrophages, and iii.) it was sustained for 3 months. Importantly, trained macrophages produced more nitric oxide, a potent antimicrobial compound, which has not been previously linked to trained immunity. Accordingly, trained macrophages better restrict leptospiral survival. Finally, we could use CL429 to train *ex vivo* human monocytes that produced more cytokines upon leptospiral stimulation. In conclusion, host-directed treatment using a TLR2/NOD2 agonist could be envisioned as a novel prophylactic strategy against acute leptospirosis.

## Author summary

Leptospirosis is a worldwide zoonotic neglected disease caused by pathogenic *Leptospira*. In addition to cattle and pets, it affects one million people per year and causes 60000 deaths. Antibiotics are efficient when given early after infection, but because the symptoms are not specific, leptospirosis is difficult to diagnose in a timely fashion. There are ~300 serovars of pathogenic *Leptospira*, which complicates the development of a universal vaccine against leptospirosis. Therefore, prophylactic treatments would be welcome. Recently, a new kind of immune memory known as “innate immune memory” or “trained immunity” has been described. It is not linked to adaptive immunity but only to innate immune cells, such as macrophages. Initial stimulation by a bacterial or fungal component can “train” macrophages to respond better but nonspecifically to a second encounter with a microbe. In this work, we show that trained immunity can be induced in mice to alleviate the symptoms of experimental acute leptospirosis. Cells from treated mice stimulated *ex vivo* by different serovars of pathogenic leptospires secreted more antimicrobial compounds and showed enhanced leptospire killing. Moreover, this treatment was able to boost human monocytes. This work presents a host-directed therapeutic approach that could be used as a novel prophylactic strategy against leptospirosis.

## Introduction

*Leptospira* belong to the phylum Spirochetes and are the etiological agents of leptospirosis, a reemerging worldwide zoonosis [1]. Pathogenic leptospires, including *Leptospira interrogans*, can infect and colonize multiple hosts, and leptospirosis constitutes a global burden affecting both human health and agricultural economics. Leptospires enter their hosts through abraded skin or mucosa and then disseminate in blood, potentially leading to renal infection [2]. Leptospirosis is underdiagnosed at its onset because of unspecific symptoms common to other viral and bacterial infections, such as fever, headaches and jaundice. Leptospirosis can cause multiorgan failure in humans and leads to a mortality rate of 6–10%. More than 300 serovars of pathogenic leptospires have been described [2]. Thus, vaccination is possible only in certain contexts, like in endemic areas when the circulating serovars are known. A few human vaccines based on inactivated whole bacteria are available, but they confer only serovar-specific and short-term protection [3]. In the absence of prophylactic drugs, preventive antibiotic therapy and wearing adapted clothes constitute the most effective prevention against this neglected disease.

Recently, we have developed a mouse model of leptospirosis using bioluminescent *L*. *interrogans* [4]. Upon intraperitoneal (IP) administration, bacteria seem to be initially controlled, but after 2 days post-infection, they are found in blood where they progressively replicate until 3 to 4 days post-infection. The bacteria are then eliminated from the blood circulation but progressively reappear in the kidneys. *L*. *interrogans* establish their niche in the proximal renal tubules, and live spirochetes excreted during urination contribute to the spread of the disease. Chronically infected rodents, such as rats and mice, are the most prominent reservoir hosts of leptospires, although other mammals, including humans can also shed *Leptospira* in urine [5].

Pattern recognition receptors (PRRs) are a subset of membrane-associated or cytosolic proteins of the innate immune system that sense microbe-associated molecular patterns (MAMPs) [6]. MAMPs are molecules that are absent in the host but essential and ubiquitously present in microbes, such as lipopolysaccharide for Gram-negative bacteria, peptidoglycan and lipoproteins. MAMPs are recognized by PRRs, which trigger antimicrobial responses, such as the production of detrimental compounds for invaders, *i*.*e*., nitric oxide (NO), reactive oxygen species (ROS) and antimicrobial peptides. Moreover, PRR activation triggers the production of pro-inflammatory cytokines and chemokines that prime the immune system, recruit phagocytes to the site of infection and initiate an adaptive response. Altogether, these responses usually result in inflammation and clearance of the invading agent [7, 8].

We have shown that *L*. *interrogans* escape some pathways of innate immune signaling. In humans, for instance, Toll-like receptor 4 (TLR4), the PRR-sensing bacterial lipopolysaccharide (LPS), cannot detect leptospiral LPS, whereas its murine counterpart is able to sense it [9]. Consistently, in opposition to wild-type (WT) mice, which are resistant to acute leptospirosis, TLR4-sensing deficiency results in sensitivity to infection with *L*. *interrogans* [10], such as in C3H/HeJ mice [11]. Additionally, we have recently shown that leptospires also escape the signaling from Nucleotide-binding Oligomerization Domain-containing protein (NOD)1 and NOD2 [12], the cytosolic receptors of muropeptides, which are the building blocks of bacterial peptidoglycan [13]. PRRs are expressed in phagocytes, and their stimulation is important for the activation of immune cells such as macrophages [7].

The role of macrophages during leptospirosis is not clear [14], and several *ex vivo* experiments have suggested that leptospires can escape the macrophage response or survive within these cells in naive hosts [15–17]. It has also been reported that NO produced by macrophages and other cells is a potent antileptospiral effector, but it is also deleterious for the host as it was linked to nephritis [18] and renal fibrosis [19]. The consequences of PRR escape by *Leptospira* on the functions of phagocytes are not yet known.

Recently, we have shown that repeated oral administration of *Lactobacillus plantarum (L*. *plantarum)* to C3H/HeJ mice alleviates acute leptospirosis and that this effect is associated with myeloid cells [20]. Therefore, we wondered whether this protection could be due to an innate immune memory effect. Indeed, recent studies have indicated that immune cells that have first been exposed to certain MAMPs respond differently than naïve cells upon a secondary encounter with the same or different MAMPs [21]. Not all MAMPs are able to induce such “memory”, and of note, different MAMPs can have positive or negative impacts for the host during a second encounter [22]. The positive or enhanced response has been coined innate immune memory (also known as trained immunity), whereas the negative effect is named tolerance [23]. Interestingly, trained immunity is a sustained long-term effect that can lead to protection against different pathogens, and it has been proposed as a vaccination strategy [24–26]. The cells involved in this response are innate immune cells such as macrophages, monocytes and natural killer cells (NK) [26]. Monocytes [27] and NK cells [28] from Bacille Calmette Guerin (BCG)-vaccinated patients show an enhanced response to mycobacterial and non mycobacterial challenges up to 3 months post-vaccination. In addition, BCG vaccination protects against unrelated lethal infection with *Candida albicans* [29] in SCID mice lacking functional B and T cells, showing that this effect is independent of the adaptive immune system. Of note, this effect can be recapitulated *ex vivo* using human monocytes trained with several MAMPs [22]. TI has been associated with PRR engagement as well as metabolic and epigenetic rewiring of innate immune cells [30–32].

The administration of *L*. *plantarum* and other probiotic bacteria demonstrates immunomodulatory properties [29, 33]. For instance, *L*. *plantarum* pretreatment protects B-cell deficient mice against lethal pneumonia virus (PMV) [34]. Moreover, using mice deficient for TLR2 and NOD2 receptors, the *L*. *plantarum* protective effect against PMV was shown to be dependent on both TLR2 and NOD2, and it was mimicked with a bi-functional TLR2/NOD2 ligand [35]. These data suggest an innate immune memory mechanism in which PRR pre-engagement enhances host antimicrobial responses upon secondary encounters. Therefore, in the present study, we hypothesized and showed that pretreatment with CL429, a chimeric TLR2/NOD2 agonist [36], could mimic the protective effect observed with *L plantarum* and alleviate the acute phase of leptospirosis due to innate immune memory.

## Materials and methods

### *L*. *interrogans* cultures

The *Leptospira interrogans* used in this work were grown in Ellinghausen-McCullough-Johnson-Harris (EMJH) medium at 28°C without agitation and diluted weekly to obtain early stationary phase growth cultures at the time of the *in vitro* and *in vivo* experiments. *Leptospira interrogans* serovars Manilae L495 (derivative bioluminescent strain MFlum1) [4], Copenhageni strain Fiocruz and Icterohaemorraghiae strain Verdun were used in this work. Bacteria were counted in a Petroff-Hauser chamber and diluted in PBS (D-PBS Lonza) before use.

### Mouse infection

Eight-week-old female or male mice of the following genotypes were used: C57BL/6J (Janvier Labs, Le Genest Saint Isle, France), Albino C57BL/6 B6(Cg)-Tyr^c-2J^/J (Charles River laboratory, Saint-Germain-Nuelles, France) and Rag2-γc (Institut Pasteur animal facility). Mice were sublethally infected with 1 or 5x10^7^ bioluminescent *L*. *interrogans* strain MFlum1 diluted in 200 μL endotoxin free D-PBS (Lonza), intraperitoneally (IP). Mice were monitored daily for weight variation and clinical signs of leptospirosis.

### *In vivo* mouse treatments

For depletion experiments, liposomes containing PBS or 5 mg/mL clodronate (Encapsula Nano Science) were injected into the peritoneal cavity in a volume of 100 μL per mouse. Liposomes were administered 3 and 1 days before sublethal IP infection with bioluminescent *L*. *interrogans*.

Priming of innate immune cells was performed in mice by IP injection of CL429 (InvivoGen) 2 and 1 week prior to infection, as previously reported by Rice *et al*., [35]. CL429 stock was solubilized in DMSO at a concentration of 5 mg/mL and further diluted in PBS. Mice were injected IP with 25 μg/mouse CL429 in 200 μL of PBS containing 2.5% DMSO. Control mice were injected with PBS containing 2.5% DMSO. Seven days after the second treatment, mice were infected with *L*. *interrogans* or euthanized by cervical dislocation to collect cells for *ex vivo* stimulation and flow cytometry analysis.

### *In vivo* imaging (IVIS)

Mice were imaged as previously described [4]. Twenty minutes after infection, mice were injected IP with 100 μL 30 mg/mL luciferin (XenoLight D-Luciferin—K^+^ Salt, Perkin Elmers) prepared in endotoxin-free PBS (Lonza) and anesthetized using an O_2_ flow rate of 1.5 L/minute and 2% atmosphere of isoflurane for 10 minutes. Under the same regimen of anesthesia, sleeping mice were transferred into the *in vivo* imaging system (IVIS Spectrum, Perkin Elmers) on a dark surface and imaged (binning 8) in automatic mode or for 5 minutes. Images were acquired for the ventral (day 0 to day 5 post-infection) or dorsal (day 8 or 9; day 15; day 30 post-infection) views.

Quantitative data were obtained in square Region of Interest (ROIs) of the complete animal, excluding the tails for ventral images (acute phase). For dorsal images, round ROIs were applied to the animals in the area corresponding to the kidneys. Quantitative data for the average light flux normalized by the time and area of ROIs were defined as light units/second/cm^2^/steradian (p/s/cm^2^/sr). Images were analyzed using Living Image software (Perkin Elmer), adjusting the photon count to 1x10^4^ to 1x10^6^ (p/s/cm^2^/sr) for all images shown.

### Ethics statement

All protocols were undertaken in compliance with EU Directive 2010/63 EU and the French regulation on the protection of laboratory animals issued on February 1, 2013. They are part of project number # 2014–0049, which was approved by the Institut Pasteur ethics committee for animal experimentation (Comité d’Ethique en Expérimentation Animale CETEA registered under #89) and was authorized under #8562 by the French Ministry of Research, the French Competent Authority.

Human blood was ordered under convention number C CPSL UNT-N° 12/EFS/134 (to JQ) from the Établissement Français du Sang, which collected informed consent from healthy volunteers.

### Harvest and stimulation of peritoneal and spleen cells

Cells were obtained from peritoneal lavage of C57BL/6 mice. Briefly, the skin was peeled off and clamped, followed by injection of 3 mL complete RPMI (RPMI GlutaMax (Gibco), 10% heat-decomplemented Foetal calf serum (FCS) (Lonza), 1 mM HEPES (Gibco), 1 mM non-essential amino acids (Gibco), 1 mM sodium pyruvate (Gibco)) supplemented with 1X antibiotic-antimycotic (Anti-Anti Gibco) into the peritoneal cavity. Next, a small incision was created in the preclamped skin and peritoneal content collected, passed through 100 μm strainer and centrifuged. Only cells from peritoneal lavage pellets lacking red blood cell contamination were used for experiments. The spleen was gently removed without damaging it and kept in complete RPMI medium. Spleens were macerated using 25G curved needles, and cells were subsequently passed through 100 μm and then 70 μm strainers. Following lysis of red blood cells using Red Blood Cells Lysis Buffer (Sigma-Aldrich), the splenocytes were washed once with complete medium. Peritoneal macrophages and spleen cells were seeded at a final concentration of 1x10^6^ cells/mL and 2x10^6^ cells/mL, respectively, and 200 μL per well was distributed into a 96-well plate (TPP). Stimulation of peritoneal or spleen cells was performed 2 to 3 h after plating. Since macrophages are adherent, peritoneal cells were washed once with PBS before stimulation to remove non-adherent cells. Cells were stimulated for 24 h after plating an equal number with 100 ng/mL *E*. *coli* LPS (EB InvivoGen) or with live *L*. *interrogans* at a multiplicity of infection (MOI) of 100.

### Bone marrow-derived macrophage (BMM) recovery and stimulation

Femurs from female and male mice were obtained after euthanasia cleaned, and the heads of the bones were removed. Two mL of medium was passed through the bones using a 21G needle to flush out the bone marrow cells. Bone marrow cells were centrifuged (300 g, 5 minutes) and treated with Red Blood Cell Lysis Buffer (Sigma-Aldrich) for 10 minutes, followed by PBS washing. The cells were enumerated, and 5x10^6^ cells were seeded in 100-cm^2^ cell culture dishes in 10 mL RPMI supplemented with 10% FCS, 1 mM sodium pyruvate, 1X Anti-Anti and 10% L929 cell supernatant. Cells were kept at 37 °C in the cell culture incubator (5% CO_2_ humidified environment) for 7 days. At day 3, 5 mL of the same medium was added. Seven days after seeding the cells, the medium was removed, and 3 mL of cell dissociation buffer (Gibco) was added. The cells were collected, centrifuged, enumerated and seeded in 96-well plates at a density of 2x10^5^ cells per well and then stimulated with different leptospires at a MOI of 1:100. At 24 h post-stimulation, nitric oxide and the cytokine dosage in the cell supernatant were assessed by the Griess reaction and ELISA, respectively. Differentiation of bone marrow cells into macrophages was assessed by flow cytometry on the day of collection using antibodies against the CD11b, F480 and CD11c markers.

### Bacterial viability assay

Peritoneal and bone marrow cells were seeded in 96-well plates at a density of 2x10^5^ cells per well in 200 μL and infected with bioluminescent *L*. *interrogans* serovar Manilae. At 24 h post-stimulation, 100 μL of the cell supernatants were distributed into white flat-bottom 96-well plate to monitor the presence of live bacteria by bioluminescence measurements in a Centro LB960 microplate luminometer (BERTHOLD), injecting 100 μL 1.5 mg/mL solution of luciferin (XenoLight D-Luciferin—K^+^ Salt, Perkin Elmers) in PBS.

### Human monocyte training

Human monocytes from blood were purified using the Pan Monocyte Isolation Kit (Miltenyi Biotec) according to the supplier’s instructions. After purification, the cells were suspended at 1x10^6^ cells/mL in RPMI without serum, and 1x10^5^ cells were seeded into a flat-bottom 96-well plate (TPP). For training, the cells were incubated for 24 h in a 5% CO_2_ atmosphere at 37 °C in the presence of the indicated agonists. Next, the cells were washed twice with prewarmed PBS, and 200 μL fresh RPMI with 10% pooled human serum (Zenbio) was added. The cells were allowed to rest for 5 days, with half of the medium replaced with fresh RPMI with 10% human serum at day 3. After resting, the cells were washed twice with prewarmed PBS and rechallenged as indicated in RPMI without serum for 24 h in a 5% CO_2_ atmosphere at 37 °C. Cell supernatants were frozen for subsequent cytokine analysis.

### NO and cytokine quantification

Nitric oxide (NO) in the cell supernatant was quantified immediately after collection using the Griess reaction. Cells supernatants were kept at -20°C until the cytokine dosage, which was performed using ELISA Duo-set kits from R&D Systems for mouse IL-1β, RANTES, IL-6, KC and human IL-6, according to the instructions provided by the supplier.

### Flow cytometry

For surface staining, a total of 2x10^5^ cells per experimental condition were washed once in a round bottom 96-well plate with MB buffer (1X PBS without Ca^2+^ and Mg^2+^; 0.5% v/v FCS, 2 mM EDTA) and resuspended in 50 μL staining buffer containing 2 μg/mL of FcBlock (anti CD16/CD32) and 1 μg/mL of antibody (see Table 1) for 20 minutes on ice. Next, 50 μL 2 μg/mL e780 fixable viability dye (eBioscience) was added for 5 minutes, followed by 50 μL of 4% PFA for fixation for 5 minutes. For intracellular staining, after fixation the cells were washed and resuspended in Inside Perm kit reagent containing 2 μg/mL of the corresponding antibody. For intracellular staining of IFN-γ, 1X Brefeldin A solution (eBioscience) was added 3 h before the collection of the cells to block the intracellular release of IFN-γ. After staining, the cells were washed 2 times with MB buffer and resuspended in 200 μL of MB buffer for acquisition. Stained cells (100 μL) were acquired (at least 50000 events for phenotyping, 25000 cells for intracellular iNOS, and 550000 cells for IFN-γ) on a MACSQuant analyzer (Miltenyi Biotec) cytometer after calibration and color compensation. Data were analyzed using FlowJo V10.

**Table 1:** Antibodies used for flow cytometry.

Target Marker (cells)	Fluorochrome	Clone	Reference	Supplier
CD11b (macrophages)	VioBlue	M1/70	48-0112-82	eBioscience
Ly6G (neutrophils)	FITC	1A8	127606	BioLegend
CD11c (dendritic cells)	PE	N418	12-0114-83	eBioscience
Ly6C (monocytes)	PerCP/Cy5.5	HK1.4	45-59-32	BioLegend
F4/80 (macrophages)	APC	BM8	123116	BioLegend
F4/80 (macrophages)	PE-CY5	BM8	15–4801	eBioscience
NK1.1 (NK cells)	VioBlue	PK136	108732	BioLegend
CD8 (T cells)	FITC	53–6.7	53-0081-82	eBioscience
CD4 (T cells)	PerCP	RM4-5	553052	eBioscience
CD3 (T cells)	PE-Cyanine7	145-2C11	25-0031-82	eBioscience
CD19 (B cells)	APC	MBI9-1	17-0191-82	eBioscience
CD16/CD32 (Fc Block)			14-0161-86	eBioscience
iNOS	PE	CXNFT	12-5920-80	eBioscience
IFN-γ	APC	XMG1.2	17-7311-82	eBioscience

## Results

### Phagocyte depletion leads to higher leptospiral loads

**Fig 1:**
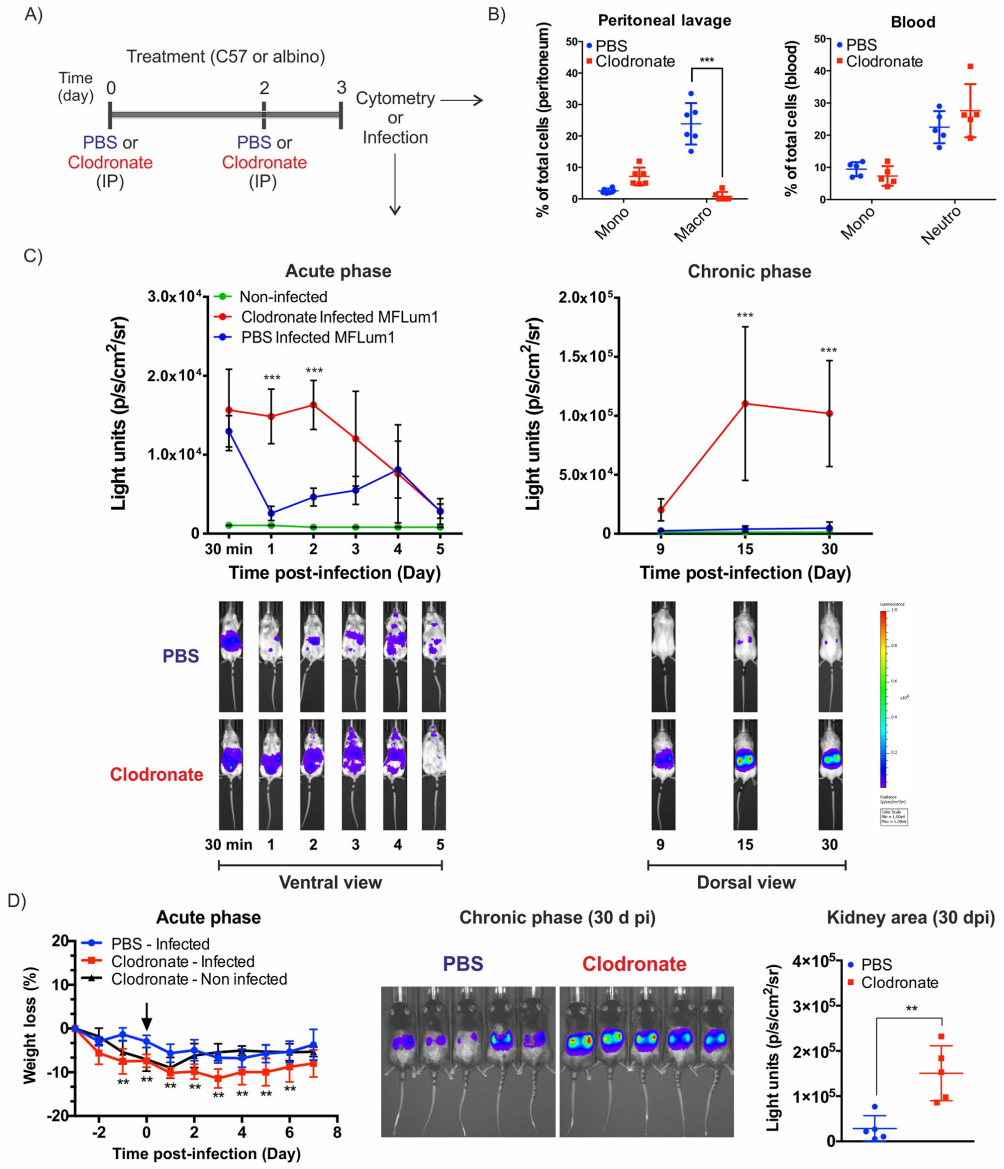
Macrophage intraperitoneal depletion leads to a nonlethal increase in leptospiral burden. A) Chronogram of macrophage depletion treatment. Mice were injected IP with 100 μL liposomes containing PBS or 5 mg/mL clodronate 3 and 1 days before cytometry analysis or IP infection. B) Flow cytometry analysis and quantification of cell subset changes in the peritoneal lavage and blood of PBS- and clodronate-treated mice (*n* = 6). Graphs represent the percentage of monocytes, macrophages and neutrophils in the peritoneum and blood (see full gating S1A Fig). C) Live imaging of albino (C57BL/6 B6(Cg)-Tyr^c-2J^/J) mice treated with PBS- or clodronate-containing liposomes and IP infected with a sublethal dose of 1x10^7^ bioluminescent *L*. *interrogans* serovar Manilae strain MFlum1. During the acute phase of infection, the mice were imaged daily (ventral view) from day 0 (30 min post-infection) to day 5. During the chronic phase from day 9, mice were imaged in the dorsal view to measure renal colonization. The lower panel shows a representative example of the follow-up of one mouse. The upper panel shows the quantification of data from n = 5 mice. The blue to red scale is proportional to the intensity of bioluminescence, reflecting the number of live leptospires. D) C57BL/6 WT mice treated with PBS- or clodronate-containing liposomes (*n* = 5) were IP infected with 5x10^7^ bacteria (MFlum1). Weight variation monitoring expressed as a percentage of the initial weight was used as a marker of acute infection. The black arrow indicates the time of infection. Live imaging during the chronic phase showing renal colonization and quantification 30 days post-infection (dpi) in the kidney area. Curve comparison was performed by 2-way ANOVA comparing PBS vs clodronate at each corresponding time point. * *p*-value < 0.05; ** *p*-value < 0.01; *** *p*-value < 0.001.

First, to delineate the potential role of phagocytic cells during *Leptospira* infection, we injected mice with clodronate liposomes to deplete phagocytes in the peritoneal cavity 3 and 1 days before infection with leptospires (Fig 1A). Phagocyte depletion was checked on the alleged day of infection in the peritoneal cavity and in blood by flow cytometry (Fig 1B) according to the gating strategy shown in (S1A Fig). We observed specific depletion of macrophages in the peritoneal cavity. In blood, no changes were observed in circulating monocytes and neutrophils (Fig 1B). Since leptospires can grow on fatty acids, we also checked that PBS and clodronate liposomes did not favor leptospiral growth *in vitro*; as expected, cultures of the bioluminescent *L*. *interrogans* MFLum1 strain were not affected by the presence of liposomes (S2A Fig).

Next, the effect of macrophage depletion on *Leptospira* infection was tested by challenging mice with a sublethal dose of bioluminescent *L*. *interrogans* (1x10^7^ bacteria/mouse), which was injected into the peritoneal cavity. Using live imaging, we observed that depletion of macrophages had a marked effect on the initial control of leptospires (Fig 1C, left panel). As previously shown, at day 1 post-infection, PBS-treated (nondepleted) infected mice had a reduced initial number of bacteria in the peritoneum. In sharp contrast, leptospiral loads in clodronate-depleted mice remained at their initial numbers until day 2 post-infection and then progressively declined. At day 5 post-infection, both groups completely controlled leptospires to the same extent. During the chronic phase, we observed a significant increase in the amount of kidney colonizing bacteria in the depleted group in comparison to mice from the PBS-treated group (Fig 1C, right panel). Of note, renal colonization in depleted mice was proportional to the extent of systemic dissemination, as previously shown [4].

To achieve higher colonization in PBS-treated mice, we performed the same experiment but with an increased sublethal dose (5x10^7^ bacteria/mouse) of bioluminescent leptospires. Although before infection clodronate treatment provoked slightly more marked weight loss than PBS-liposomes, after infection clodronate-treated mice lost more weight than their PBS counterparts (Fig 1D). One month post-infection, live imaging of the kidney revealed that PBS-treated mice had the expected levels of colonization and that depleted mice presented increased kidney colonization (Fig 1D).

These results indicate that peritoneal macrophages play a role in the initial control of *L*. *interrogans* administered IP in the mouse model. The protective role of macrophages is partial since no lethality was observed upon depletion. These data also suggest that training of macrophages could potentially have a positive impact on leptospirosis outcomes.

### Treatment of mice with a TLR2/ NOD2 agonist alleviates acute leptospirosis

We have recently shown that oral treatment with *Lactobacillus plantarum* can alleviate acute leptospirosis in mice through a myeloid cell-mediated effect [20]. Interestingly, Rice *et al*., [35] have shown that the *L*. *plantarum* effect can be mimicked with CL429, a synthetic bifunctional TLR2/NOD2 agonist corresponding to murabutide, a NOD2 agonist that is linked to Pam2cys, a TLR2 agonist [36]. Therefore, using TLR2 or NOD2-transfected HEK293T NF-κB reporter cell lines, we first checked the specificity of our strain of *L*. *plantarum* (strain 256). As expected, we found that both CL429 and *L*. *plantarum* 256 triggered the activation of TLR2 and NOD2 (S2B Fig). We also checked that CL429, which was reported not to affect mice [36], also did not affect *in vitro* cultures of leptospires (S2A Fig). Hence, we hypothesized that CL429 might, such as *L*. *plantarum*, exert a protective effect upon infection with *L*. *interrogans*.

**Fig 2:**
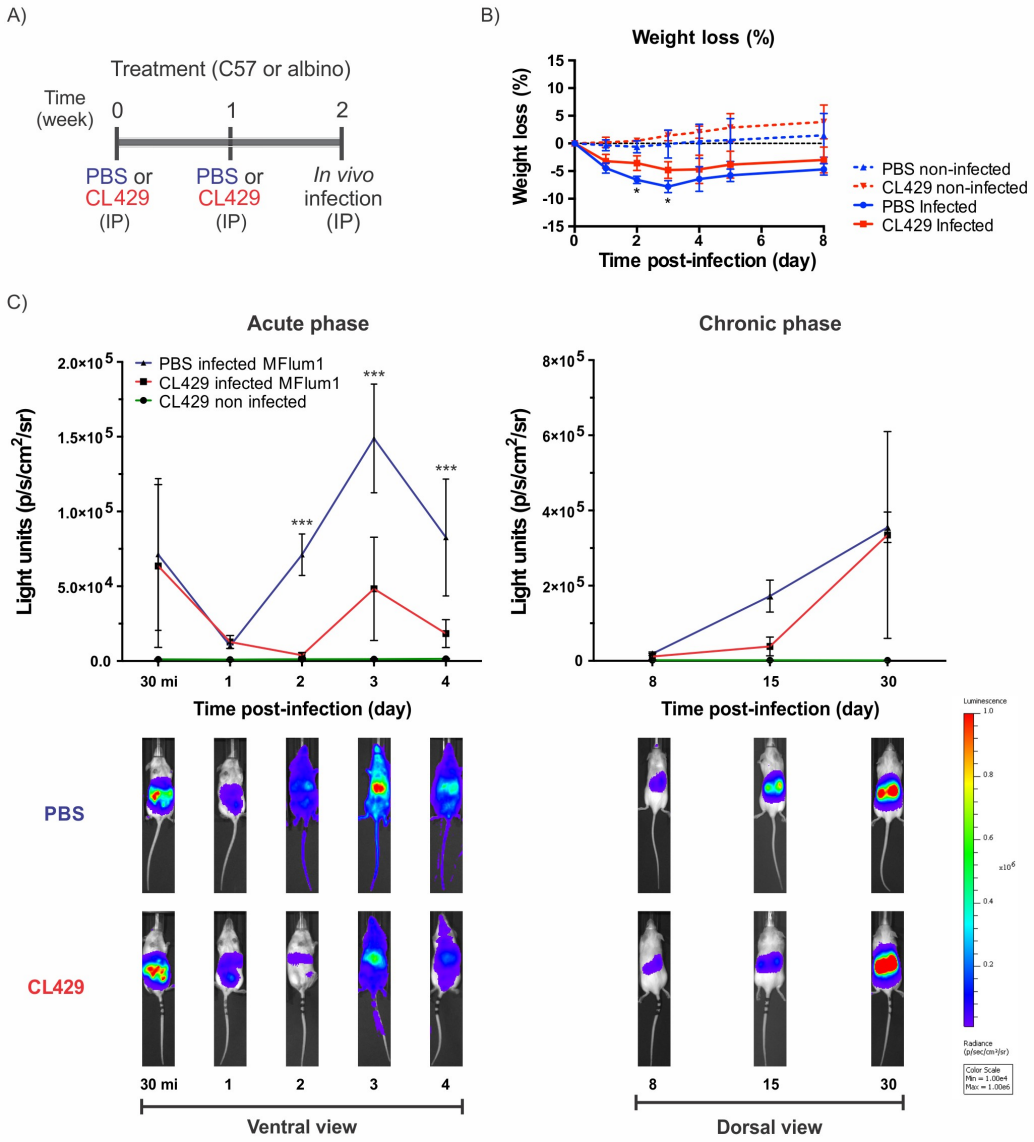
Treatment with CL429 enhances leptospiral clearance in the acute but not the chronic phase of infection. A) Chronogram of the experiment. C57BL/6 WT mice were injected IP with 200 μL PBS containing 2.5% DMSO (vehicle) or 25 μg CL429 2 and 1 weeks before IP infection. B) C57BL/6 WT treated mice (n = 5) were IP infected with 5x10^7^ leptospires, and weight variation monitoring, expressed as a percentage versus of the weight on the day of infection, was used to follow the severity of acute infection. C) Live imaging of albino (C57BL/6 B6(Cg)-Tyr^c-2J^/J) treated mice IP infected with a sublethal dose of 5x10^7^ bioluminescent *L*. *interrogans* serovar Manilae bioluminescent strain MFlum1. During the acute phase of infection, mice were daily imaged (ventral view) from day 0 (30 minutes post-infection) to day 4. During the chronic phase from day 8, mice were imaged in the dorsal view to measure renal colonization. The lower panel shows a representative example of the follow-up of one mouse. The upper panel shows the quantification of data from n = 4 mice. The blue to red scale is proportional to the intensity of bioluminescence, reflecting the number of live leptospires. Data represent 1 of 2 independent experiments (n = 4 or 5). Curve comparison was performed by 2-way ANOVA comparing PBS vs clodronate at each corresponding time point. * *p*-value < 0.05; ** *p*-value < 0.01; *** *p*-value < 0.001.

We treated C57/BL6 mice IP with CL429 at 2 weeks and 1 week before IP infection with a sublethal dose of the bioluminescent *L*. *interrogans* MFLum1 strain (Fig 2A). Infected CL429-treated mice showed slight but significant reduced weight loss compared with PBS-infected controls, which could be a clinical sign of enhanced bacterial control (Fig 2B). We repeated this experiment and tracked the infection in CL429-treated or control albino mice by live imaging of bioluminescent *Leptospira* (Fig 2C). We observed a similar control of leptospires at day 1 post-infection between the two groups (Fig 2C). However, from day 2 to day 4 post-infection, we observed a marked reduction of the bacterial burden in treated mice, corresponding to a lack of dissemination from the peritoneal cavity, whereas control mice had systemic dissemination of leptospires (Fig 2C, lower left panel). We also evaluated kidney colonization during the chronic phase and observed a reduction in the kidney load at day 15 post-infection in CL429-treated mice, although at day 30 post-infection, the kidney loads were equivalent in both groups (Fig 2C, right panels).

In conclusion, CL429 treatment alleviated the acute phase of infection, although it did not result in kidney clearance. These findings further substantiate what we observed previously *with Lactobacillus plantarum*-treated C3H/HeJ mice [20].

### Treatment of mice with a TLR2/NOD2 agonist leads to an enhanced local antileptospiral response *ex vivo*

**Fig 3:**
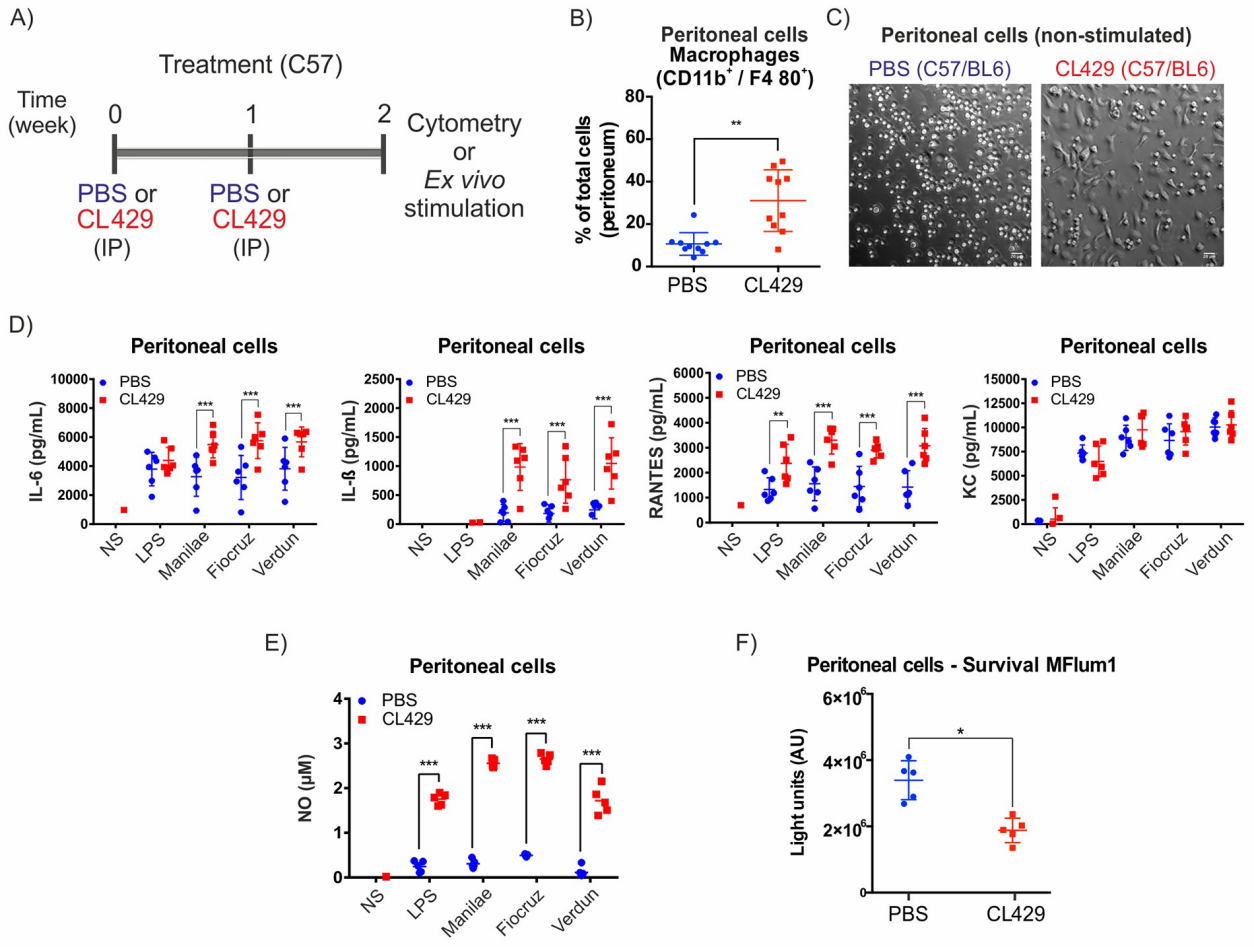
Treatment with CL429 enhances *ex vivo* antibacterial responses. A) Chronogram of the experiment. C57BL/6 WT mice were injected IP with 200 μL PBS containing 2.5% DMSO (vehicle) or 25 μg CL429 2 and 1 weeks before the collection of peritoneal cells for cytometry or *ex vivo* stimulation. Cells were plated, and adherent cells were exposed for 24 h to 100 ng/mL *E*. *coli* LPS or live *Leptospira interrogans* serovar Manilae, Fiocruz or Verdun at a MOI of 100. B) Flow cytometry analysis showing the percentage of macrophages (CD11b^+^/F4/80^+^) recovered in peritoneal lavages from treated (CL429) or control (PBS) mice. The plot corresponds to 2 independent experiments pooled with n = 5 mice in each group. C) Representative phase contrast microscopy image of adherent peritoneal cells seeded in 96-well plates at 3 h post-collection (non stimulated (NS) cells). D) Pro-inflammatory cytokine (IL-6, IL-1β) and chemokine (RANTES, KC) production determined by ELISA 24 h post-stimulation in the supernatant of peritoneal cells. Data are representative of 4 independent experiments (n = 4 to 6 mice per group, treated individually). E) Nitric oxide (NO) production in peritoneal cells 24 h after stimulation assessed by the Griess reaction. Data are representative of 4 independent experiments (n = 4 to 6 mice per group, treated individually). F) Peritoneal macrophages were exposed to bioluminescent *L*. *interrogans* serovar Manilae MFlum1 strain at MOI of 100. Bioluminescence was measured 24 h post-stimulation in 100 μL culture supernatant to assess the number of live leptospires. Data are representative of 2 independent experiments (*n* = 5). Statistical analysis was performed by 2-way ANOVA comparing PBS vs CL429 for each stimulation. For macrophage phenotyping (B) and survival (F), statistical analysis was performed by the unpaired *t*-test. * *p*-value < 0.05; ** *p*-value < 0.01; *** *p*-value < 0.001.

To understand whether CL429 treatment, which helped mice to control *Leptospira*, could have boosted the macrophage response as expected for innate immune memory, mice were treated with CL429, but instead of being infected, mice were sacrificed to collect peritoneal cells for *ex vivo* stimulation with pathogenic leptospires (Fig 3A).

Two weeks after the first CL429 injection, we checked the peritoneal composition by flow cytometry and observed an increased percentage of macrophages among total peritoneal cells upon CL429 treatment (Fig 3B), as well as a reduction in T cells (S1B Fig), although the proportions of other cell types were not affected. Since morphological changes have been associated with a trained cellular state [22], after plating an equal number of peritoneal cells and washing to discard non-adherent cells, we examined non stimulated cells by bright field microscopy and observed significant morphological changes in cells from CL429 pretreated mice compared with the PBS controls (Fig 3C). An enhanced pro-inflammatory response has been defined as one of the key aspects of trained immunity. Therefore, we checked the production of pro-inflammatory mediators at 24 h post-stimulation with LPS or live leptospires by ELISA. We observed a significant increase in the production of IL-6, IL-1β, and RANTES after stimulation with 3 different pathogenic serovars of *L*. *interrogans* (Manilae, Verdun and Fiocruz), but the amount of KC produced by these cells was not affected by the treatment (Fig 3D). Of note, the lack of IL-1ß production after LPS stimulation was expected, since IL-1ß secretion is tightly regulated in mice and requires two signals that we have previously shown to be provided by live *L*. *interrogans* through TLR2/4 and the NLRP3 inflammasome [37]. Importantly, cytokine production upon stimulation with the 3 different pathogenic serovars of leptospires was equally enhanced. Finally, since murine macrophages produce nitric oxide (NO), which has been described as a potent antileptospiral mediator as well as a key pro-inflammatory signature, we tested NO production in peritoneal cells 24 h post-stimulation. Interestingly, we found that peritoneal cells from CL429-treated mice showed enhanced NO production when rechallenged with leptospires or LPS, although strikingly, cells treated with PBS produced minimal amounts of NO upon *L*. *interrogans* stimulation (Fig 3E). Finally, we tested the ability of peritoneal cells to kill bioluminescent leptospires at 24 h post infection. We found fewer live leptospires in peritoneal cells of CL429-treated mice compared with the level of bacteria in cells of PBS-treated mice (Fig 3F). Altogether, these *ex vivo* data suggest that CL429 priming enhances the ability of macrophage to combat leptospires.

### TLR2/NOD2-associated antibacterial response is mediated mainly by myeloid cells

**Fig 4:**
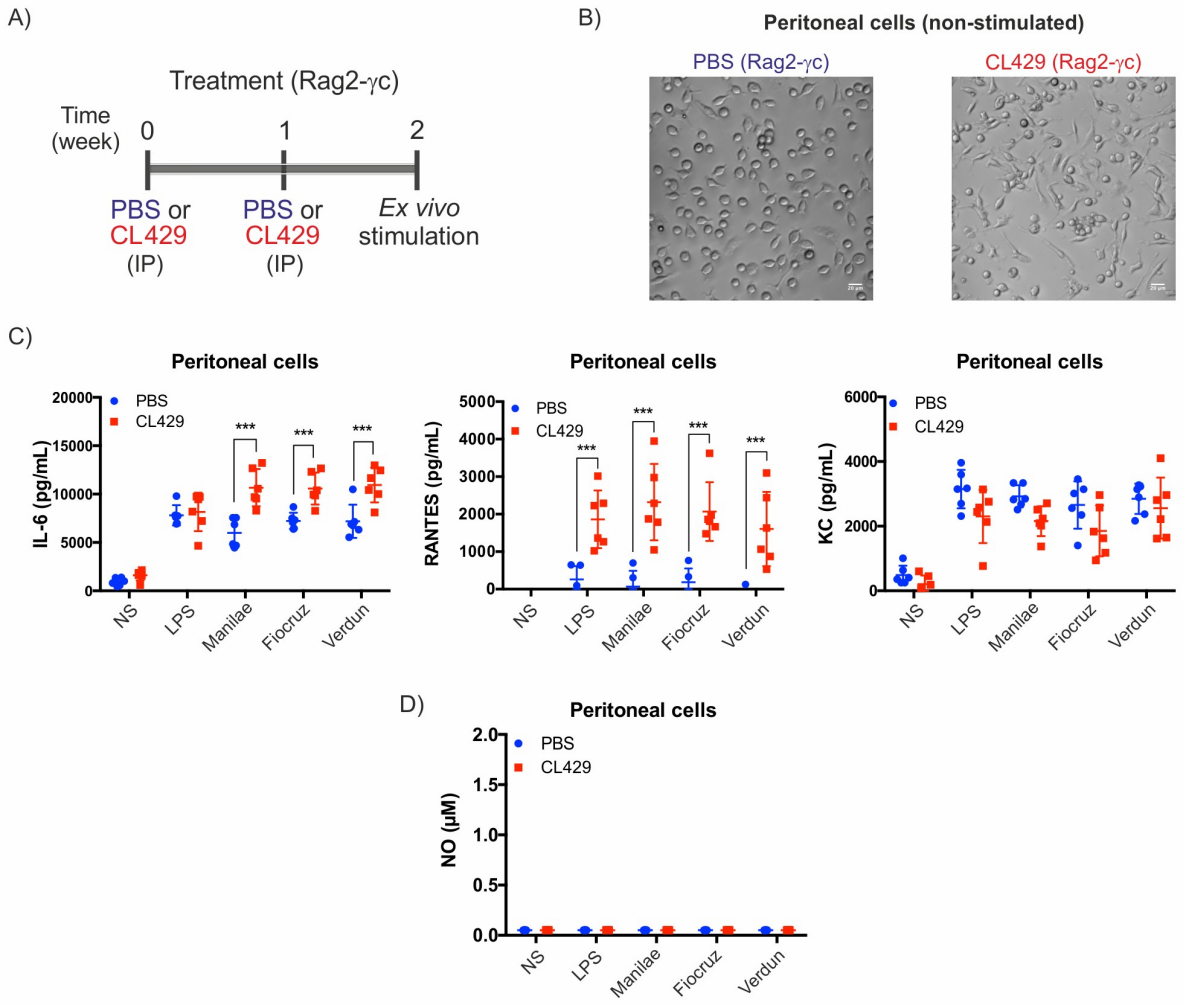
Myeloid cells are responsible for CL429-induced protection in the peritoneal cavity. A) Chronogram of the experiment. Rag-2γc mice were injected IP with 200 μL PBS containing 2.5% DMSO (vehicle) or 25 μg CL429 2 and 1 weeks before the collection of peritoneal cells for *ex vivo* stimulation. Cells were plated and exposed for 24 h to *E*. *coli* LPS (100 ng/mL) or *Leptospira interrogans* serovar Manilae, Fiocruz or Verdun, at MOI of 100. B) Representative phase contrast microscopy image of non stimulated peritoneal cells seeded in 96-well plates at 3 h post-collection. C) Pro-inflammatory cytokine (IL-6) and chemokine (RANTES and KC) production determined by ELISA in the supernatant of cells 24 h post-stimulation. Data are representative of 2 independent experiments (n = 6 mice per group, treated individually). Statistical analysis was performed by the unpaired *t*-test comparing PBS vs CL429. D) Nitric oxide (NO) production in peritoneal cells at 24 h after stimulation assessed by the Griess reaction. Data are representative of 2 independent experiments (n = 6 mice per group, treated individually). Statistical analysis was performed by 2-way ANOVA comparing PBS vs CL429 for each secondary stimulation. * *p*-value < 0.05; ** *p*-value < 0.01; *** *p*-value < 0.001.

Trained immunity has been shown to be independent of the adaptive immune response [26]. Therefore, to understand whether the CL429-mediated effect is independent of lymphoid cells, we used Rag2-γc mice deficient in B, T and NK cells. We treated these mice with CL429 as previously performed with WT mice (Fig 4A) and collected the cells for *ex vivo* functional and microscopy analysis.

After CL429 treatment, we observed a change in morphology of plated peritoneal cells from Rag2-γc mice (Fig 4B), as was observed with wild-type (WT) mice. We observed enhanced cytokine production of IL-6 and RANTES, but not KC, after stimulation with 3 leptospiral strains (Fig 4C), reproducing the effect observed in immune-competent mice. However, we did not find any NO production in peritoneal cells from PBS- or CL429-treated Rag2-γc mice (Fig 4D).

These results obtained using mice deficient for B, T and NK cells indicate that our observed phenotype on enhanced cytokine production and morphology is driven by innate myeloid cells and is independent of the adaptive T and B cell compartment, which is consistent with trained immunity.

### CL429 treatment leads to systemic effects on splenic and bone marrow cells

**Fig 5:**
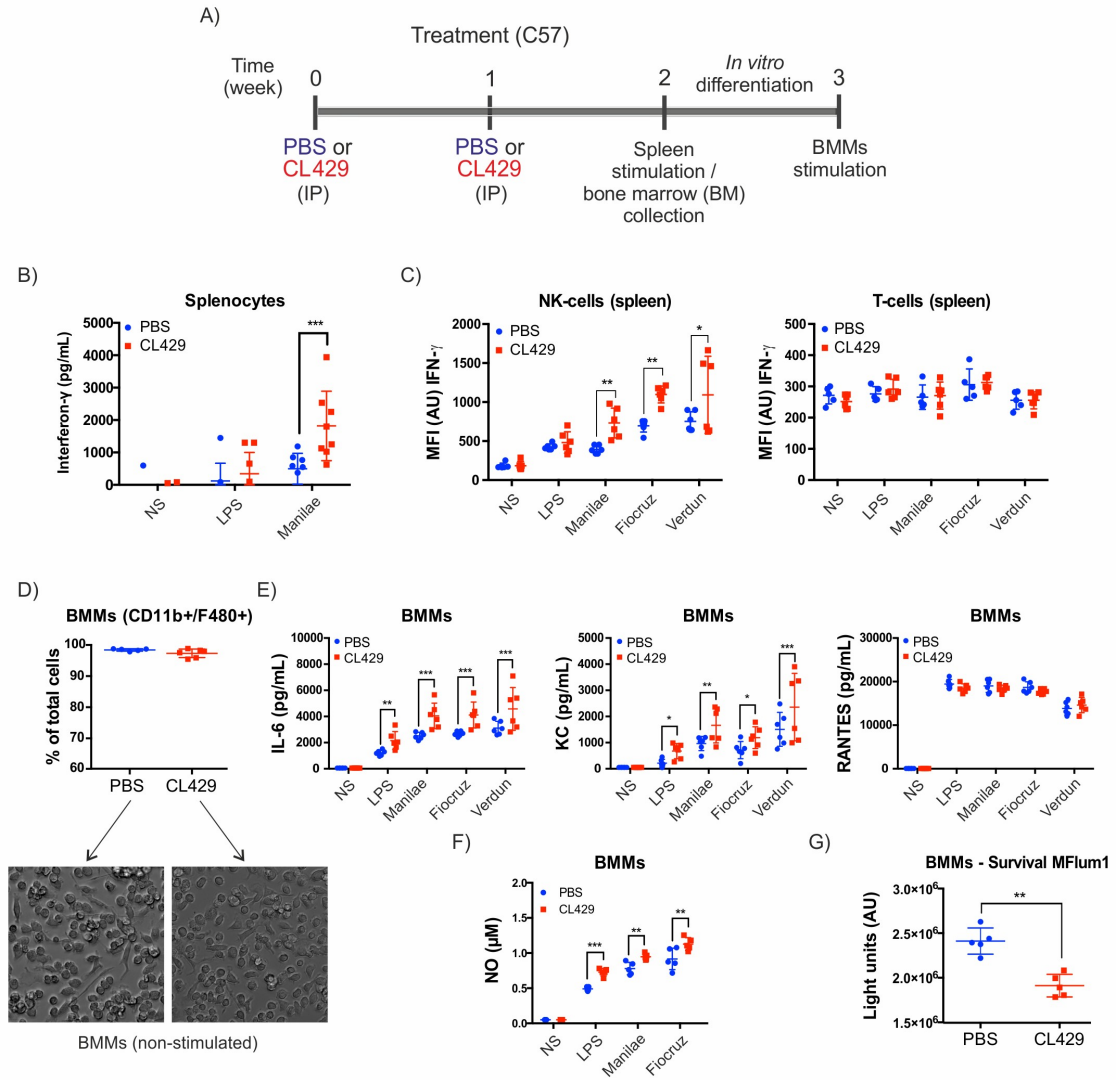
CL429 treatment leads to a systemic enhanced antileptospiral response. A) Chronogram of the experiment. C57BL/6 WT mice were injected IP with 200 μL PBS containing 2.5% DMSO (vehicle) or 25 μg CL429 2 and 1 weeks before the collection of splenic and bone marrow cells for cytometry staining, macrophage differentiation and *ex vivo* stimulation. Splenic cells were plated and exposed for 24 h to 100 ng/mL *E*. *coli* LPS or live *Leptospira interrogans* serovar Manilae, Fiocruz or Verdun at a MOI of 100. Bone marrow cells were differentiated into bone marrow-derived macrophages (BMMs) for 7 days, plated and stimulated with LPS or live leptospires. B) Interferon(IFN)-γ IFN production in the supernatant of splenic cells 24 h post-stimulation assessed by ELISA. Data correspond to 2 independent experiments pooled with n = 4 mice per group, treated individually. C) Flow cytometry analysis of IFN-γ produced by splenic NK and T cells at 24 h post-stimulation. Data correspond to one experiment with n = 6 female mice. D) Flow cytometry analysis of BMMs 7 days post-differentiation of bone marrow cells (upper panel) and representative microscopy images of BMMs showing no major morphological differences in BMMs from PBS- or CL429-treated mice (lower panel). E) Pro-inflammatory cytokine (IL-6) and chemokine (RANTES and KC) profiles determined by ELISA on the supernatant of cells 24 h post-stimulation. F) Nitric oxide (NO) production by BMMs at 24 h after stimulation assessed by the Griess reaction. G) Survival of *L*. *interrogans* serovar Manilae bioluminescent strain MFlum1 in contact with BMMs assessed by bioluminescence measurement in culture 24 h post-infection. (D-G) Data are representative of 2 independent experiments with n = 5 mice per group, treated individually. Statistical analysis was performed by the unpaired *t*-test comparing PBS vs CL429 for each secondary stimulation. * *p*-value < 0.05; ** *p*-value < 0.01; *** *p*-value < 0.001.

Since the observed peritoneal effect was localized to the site of injection, we aimed to understand whether CL429 treatment could also have systemic consequences. First, we assessed if CL429 injection *in vivo* (Fig 5A) led to changes in blood or splenic cell populations and using flow cytometry, we observed no significant changes upon CL429 administration to mice (S1C and S1D Fig). Next, the production of inflammatory mediators was assessed in splenocytes from PBS- or CL429-treated mice, and we observed by ELISA significantly enhanced production of interferon-γ (IFN-γ) upon *Leptospira* infection in comparison with the PBS-treated counterparts (Fig 5B). Next, using flow cytometry, we examined which cell type produced IFN-γ in splenic cells. We observed significantly increased IFN-γ secreted by splenic NK cells upon stimulation with different pathogenic serovars, although no induction of IFN-γ was observed in T cells under our conditions (Fig 5C).

Additionally, we tested whether progenitors from the bone marrow could also have been primed by CL429 treatment. At 2 weeks post-CL429 or -PBS treatment, we collected bone marrow cells and differentiated them into bone marrow macrophages (BMMs) (Fig 5A). First, we assessed whether the differentiation process of bone marrow cells led to macrophages. We observed that after a week of differentiation, more than 95% of the cells were macrophages (CD11b^+^/F4/80^+^) (Fig 5D). In addition, we did not observe any major morphological differences in BMMs by microscopy when comparing BMMs obtained from PBS and CL429-treated mice. We then studied the secretion of pro-inflammatory compounds by BMMs. Upon restimulation with different leptospiral serovars, we observed increased production of IL-6 and KC but the same production level of the chemokine RANTES (Fig 5E). Finally, we observed increased production of antimicrobial NO upon stimulation with different pathogenic serovars (Fig 5F). Since NO has been associated with leptospiral killing, we assessed bacterial survival using a bioluminescent Manilae strain. We observed enhanced bactericidal activity in BMMs from CL429-treated mice compared with PBS-treated mice (Fig 5G), as we observed in peritoneal cells.

Altogether, these results indicate that treatment with CL429, a TLR2 and NOD2 agonist, leads to increased production of cytokines and antibacterial mediators *ex vivo*, in the peritoneal cavity, the bone marrow and the spleen, which are remote from the site of infection. This local and systemic effect is consistent with trained immunity. Therefore, we wondered whether the BMMs originating from the CL429-treated mice could express more PRRs, as has been previously described for BCG inducing trained immunity and TLR4 upregulation [38]. We measured the expression of NOD1, NOD2 and TLR4 in BMMs by qRT-PCR but did not detect an upregulation of those receptors in non stimulated BMMs derived from trained mice compared with PBS-treated mice (S3 Fig). Upon restimulation with leptospires (Manilae serovar), TLR2 mRNA expression was clearly upregulated compared with non-stimulated cells, although TLR4 and NOD2 were not or were only modestly upregulated, respectively. However, no difference was observed between stimulated BMMs harvested from CL429- or PBS-treated mice.

### CL429 treatment leads to long-lasting phenotypic and functional changes in cells

**Fig 6:**
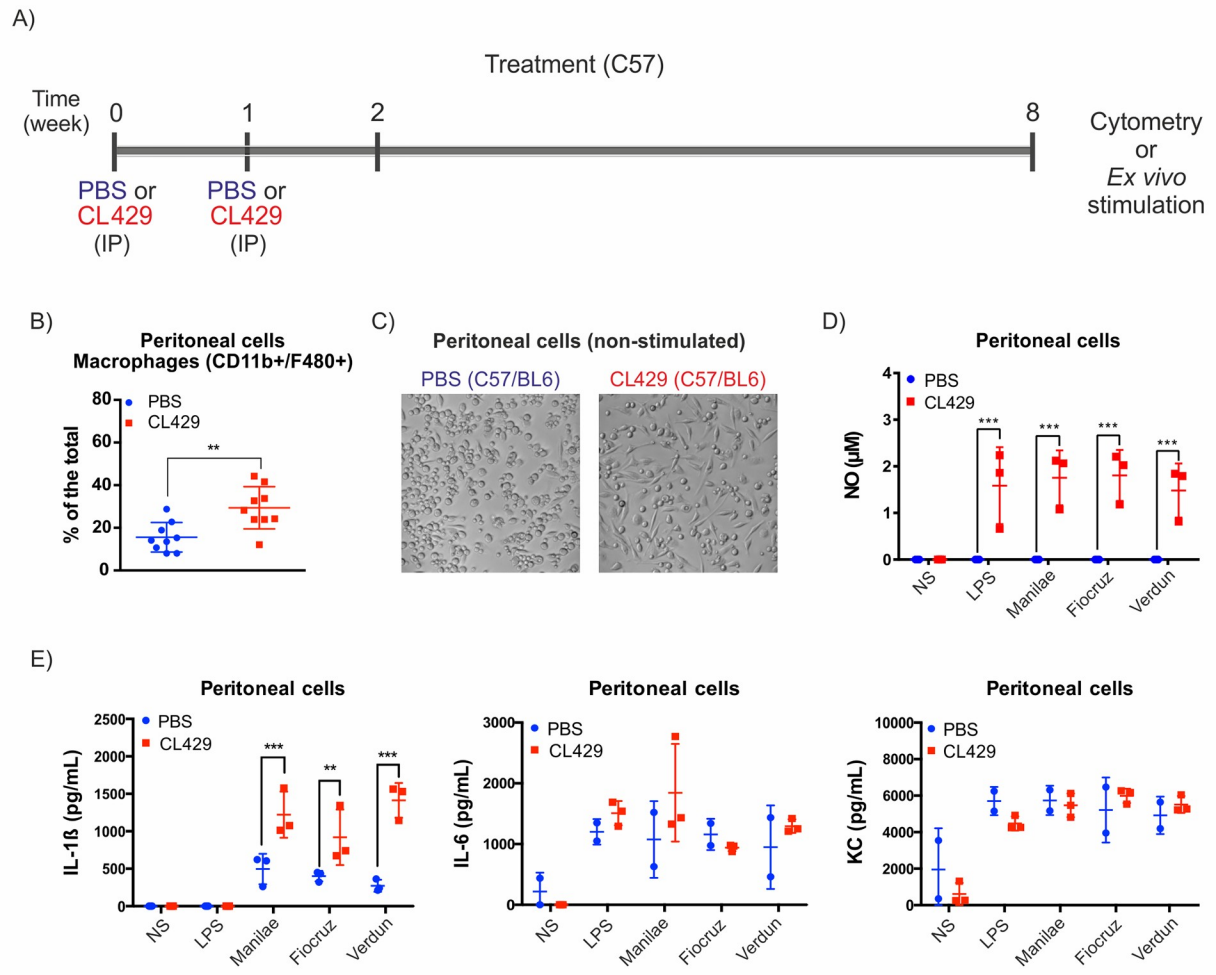
CL429 treatment leads to a long-term enhanced antileptospiral response. A) Chronogram of the experiment. C57/BL6 WT mice were injected IP with 200 μL PBS containing 2.5% DMSO (vehicle) or 25 μg CL429 8 weeks before the collection of peritoneal cells for *ex vivo* stimulation. Cells were plated and exposed for 24 h to *E*. *coli* LPS (100 ng/mL) or live *L*. *interrogans* serovar Manilae, Fiocruz or Verdun, at MOI of 100. B) Flow cytometry analysis of the peritoneal lavages showing the proportions of (CD11b^+/^F4/80^+^) macrophages from CL429-treated and PBS-treated mice 8 weeks post-treatment. C) Representative phase contrast microscopy images of non stimulated peritoneal cells from PBS- and CL429-treated cells seeded in 96-well plates 3 h post-collection. D) Nitric oxide (NO) production in peritoneal cells 24 h after stimulation assessed by the Griess reaction. E) Pro-inflammatory cytokines (IL-1ß, IL6) and chemokine (KC) production determined by ELISA 24 h post-stimulation in the supernatant of peritoneal cells. Data are representative of 2 experiments in (n = 3 or 4) mice. Statistical analysis was performed by 2-way ANOVA comparing PBS vs CL429 for each stimulation. For macrophage phenotyping (B), statistical analysis was performed by the unpaired *t*-test. * *p*-value < 0.05; ** *p*-value < 0.01; *** *p*-value < 0.001.

To assess whether the effects of CL429 could be sustained over time, we repeated the experiments (with two CL429 injections, Fig 6A) and collected peritoneal, splenic and bone marrow cells at 8 weeks or 3 months post-treatment rather than 2 weeks after the first injection of CL429.

By flow cytometry, we observed an increased proportion of macrophages in the peritoneum of CL429-treated compared with PBS control mice (Fig 6B), although we did not detect other changes in the peritoneal cavity (S4A Fig). Interestingly, this increase at 8 weeks was similar to the one observed at 2 weeks after the first injection of CL429. Moreover, we also observed the characteristic morphological changes in peritoneal cells from CL429-treated mice that we observed at 2 weeks post-treatment (Fig 6C). Next, LPS and 3 strains of live *L*. *interrogans* were used to stimulate the cells for 24 h. Enhanced production of pro-inflammatory mediators (NO and IL-1ß) was observed (Fig 6D and 6E), and the cellular response to leptospiral challenge was similar in different strains and comparable to the response observed at 2 weeks after the first CL429 injection. However, if the KC response was unchanged, IL-6 cytokine was not as enhanced as previously observed 2 weeks after treatment (Fig 6E).

To determine whether the enhanced cytokine and NO production observed after *ex vivo* stimulation were due to an increased number of macrophages or if CL429 treatment enhanced the reactivity of those cells, we detached the adherent cells at 24 h post-infection and analyzed their phenotype by flow cytometry as well as the expression of iNOS, the enzyme responsible for NO production. Consistent with our results using peritoneal lavages, we observed an enhanced number of macrophages (CD11^+^/F4/80^+^). Interestingly, if the number of macrophages among the plated peritoneal cells was higher in the CL429-treated mice (S4B Fig), the expression of iNOS was strikingly enhanced in the CL429-treated macrophages infected with LPS or leptospires, compared to PBS-treated infected cells, whereas treated noninfected cells showed the same basal expression (S4C Fig). This result suggests that CL429 treatment not only enhances the number of macrophages in the peritoneal cavity but also induces innate immune memory of macrophages. The enhanced expression of iNOS in macrophages was comparable upon stimulation with the 3 strains of leptospires (S4D Fig).

Consistent with the results obtained at 2 weeks post-treatment, although no change in number was observed (S5A and S5B Fig), splenic NK cells from mice treated 3 months prior with CL429 exhibited more intracellular IFNγ than their PBS counterparts (S5C and S5D Fig). In addition, BMMs from mice 3 months post-treatment presented increased levels of IL-6 and KC and NO, although the levels of RANTES were not different (S6 Fig).

Altogether, these results indicate that injection of CL429 leads to profound modifications to splenic NK and macrophages in the environment of the peritoneal cavity as well as in their progenitors in the bone marrow and that the increased response to subsequent challenges with leptospires or *E*. *coli* LPS persists for at least 3 months.

### CL429 pretreatment of human primary monocytes enhances the IL-6 response against *Leptospira*

**Fig 7:**
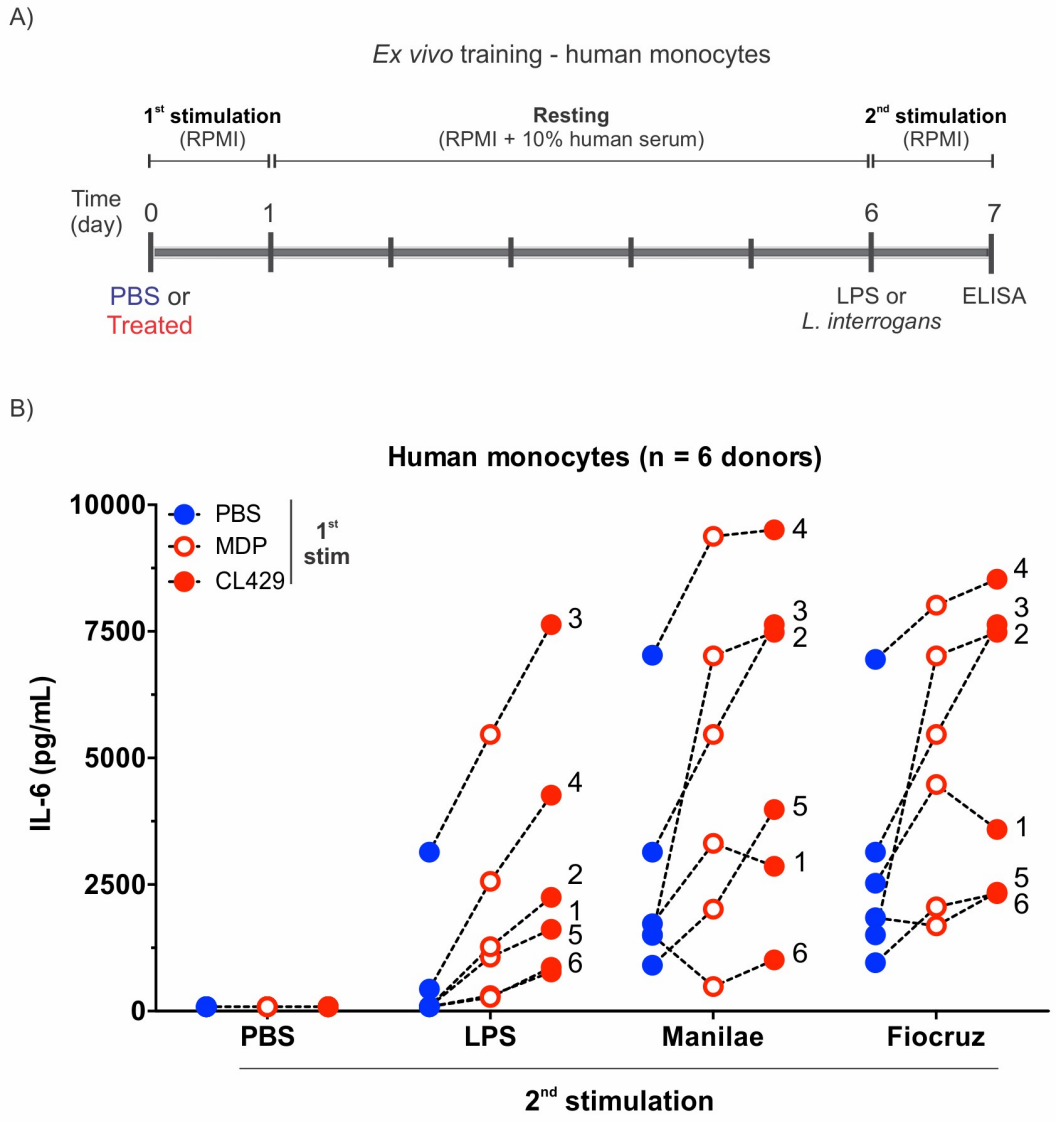
Training of human primary monocytes with CL429 enhances the IL-6 pro-inflammatory cytokine response. A) Chronogram of *in vitro* training of human monocytes isolated from healthy human blood. B) Comparative IL-6 profiles for n = 6 individual donors (performed in 3 independent experiments) following different initial stimulations with PBS, MDP or CL429 each at 0.1 μM. The second stimulation was performed with PBS as a control, LPS (100 ng/mL) or live leptospires (serovars Manilae and Fiocruz) at MOI of 100. Each number corresponds to an individual donor. Each dot represents a training condition for a particular donor, and the graphed value corresponds to the average of 4 technical replicates.

Trained immunity induced by PRR ligands can be recapitulated in humans primary monocytes *ex vivo* [22]. To understand whether CL429 could induce innate immune memory in human monocytes and increase their responsiveness towards leptospires, human primary monocytes isolated from healthy donors were individually seeded in plates and stimulated for 24 h with PBS or treated with CL429 or MDP, the NOD2 agonist used as a positive control of trained immunity [22, 38]. After this first stimulation or “training”, the cells were allowed to rest for 5 days and then were restimulated for 24 h with LPS or live pathogenic *L*. *interrogans* strains (Fig 7A). IL-6 measured by ELISA in the supernatants was used as a read-out to determine their inflammatory status (Fig 7B). Several doses of MDP and CL429 were tested. A concentration of 0.1 μM was chosen since it did not affect cell viability after the second stimulation (S7 Fig).

Even though the IL-6 response within donors was very variable (Fig 7B), we observed training for both MDP and CL429 in 6/6 independent donors after LPS and Fiocruz restimulation and in 5/6 donors after Manilae stimulation. Strikingly, in all donors tested except donor 1, CL429 treatment enhanced IL-6 production to a greater extent than the positive control MDP. These results show that CL429 can boost human monocytes and enhance their responsiveness to *Leptospira* infection.

## Discussion

In this study, we explored the role of macrophages in a mouse model of leptospirosis. On the one hand, we used a depletion approach with a treatment with clodronate liposomes that suggests a role for macrophages in the early stages of leptospiral infection. On the other hand, we showed that pretreatment with CL429, a bifunctional TLR2/NOD2 ligand, induces protection against leptospiral infection. Pretreatment with CL429 modified the environment of the peritoneal cavity with macrophage enrichment and boosted the responses of macrophages against a subsequent leptospiral infection. These effects in mice correspond to the described trained immunity characteristics and were recapitulated in human monocytes. These results suggest that CL429 pretreatment could be used to boost human and animal cellular responses, which could potentially open up prophylactic strategies to control acute *L*. *interrogans* infection.

Our clodronate macrophage depletion findings indicate that the lack of these cells influenced both the acute and chronic phases of leptospirosis, although the depletion treatment had only been done before infection. First, we consistently observed an initial decrease of bacteria at day 1 post-infection in WT mice [4], which was not present upon IP clodronate depletion treatment. Therefore, we attribute this initial decline in bacterial burden to a protective effect of peritoneal macrophages, which killed some leptospires, as we showed *in vitro*, but not in sufficient numbers to avoid systemic infection. Although IP depletion of macrophages had no lethal effect, it led to enhanced bacteremia during the acute phase and to increased bacterial colonization of the kidneys. This result is consistent with the findings of Isogai *et al*., using silica intravenous (IV) depletion, in which bacteria were more prominent in IV depleted mice during the acute phase, without a lethal outcome [39]. Since we did not observe monocyte depletion in the blood, it is unlikely that the IP clodronate treatment could have also depleted the macrophages in the renal tissue. This result suggests, as was previously observed [4], that early control of bacteria predicts the future extent of renal colonization. It is also in line with the recent study by Ferrer *et al*., [40], based on a clodronate IP depletion regimen that was maintained after infection with *L*. *interrogans* serovar Copenhageni strain Fiocruz. They also showed that macrophages controlled the leptospiral burden in mice. Since macrophage depletion increased the renal bacterial load, we may also conclude, in accordance with Ferrer’s study, that *L*. *interrogans* serovar Manilae was not transported as cargo by macrophages to the mouse kidney, as has been previously suggested in zebrafish using the Fiocruz strain [41].

Upon injection of CL429, the peritoneal cavity underwent a modification in its immune cells composition. Even though we did not observe any major changes in the cell populations in other compartments, there was an increased number of macrophages in the peritoneal cavity. This increase was specific since it did not affect the resident population of other cell types such as B or NK cells, although compensation was provided by a slight decrease in the percentage of T lymphocytes. This change in macrophages in the peritoneal cavity and the enhanced inflammatory response of CL429-treated peritoneal cells were sustained at 3 months post-treatment, indicating that the treatment had a long-term effect. Using CL429-treated WT or Rag-2γc mice (lacking T, B and NK cells), we obtained similar enhanced cytokine responses of peritoneal cells after *ex vivo* leptospiral stimulation, showing a major innate immune contribution of macrophages to this phenotype. Altogether, these results indicate that the injection of CL429 led to a new homeostasis in the peritoneal cavity with long-lasting functional changes in macrophages to render them more responsive to leptospires and other stimuli. Interestingly, we demonstrated that the effect of CL429 treatment was not only local but also had systemic effects on splenic NK cells and on bone marrow myeloid progenitors, which maintained their “training” after *ex vivo* differentiation, as recently described for BCG and ß-glucan [42, 43]. Future questions will address whether the trained peritoneal macrophages are resident cells and whether CL429 treatment increases their numbers or if they emanate from the bone marrow and are then recruited from the blood to be established in the peritoneum. For instance, infection with murine herpesvirus 4 leads to the replacement of lung macrophages by inflammatory monocytes, thereby conferring protection against house dust mite-induced experimental asthma [44].

Intraperitoneal administration of CL429 alleviated acute leptospiral infection in mice. The bacterial burden in mice was reduced due to the enhanced killing ability of macrophages. This treatment delayed but did not reduce kidney colonization at one month post-infection. These results resemble the observation made for C3H/HeJ mice after *Lactobacillus plantarum* oral treatment [20]. In our model, only two IP doses, compared with 30 oral doses of *L*. *plantarum* in [20], were sufficient to alleviate leptospirosis in a similar fashion. Here we showed that the protective effect of CL429 was due to enhancement of the macrophage number and functions mimicking trained immunity or an innate immune memory phenotype. Interestingly, recruitment of macrophage-like cells has also been observed in the kidneys of *Lactobacillus plantarum*-treated mice, at 15 days post-infection with leptospires [20]. By extension, we may infer that the positive effect on acute leptospirosis previously observed with oral treatment with *Lactobacillus plantarum*, which is agonist of both TLR2 and NOD2, was probably due to a trained immunity effect. Regular consumption of probiotics such as *Lactobacillus plantarum*, a generally recognized as safe (GRAS) organism, could be envisioned as a prophylactic treatment to avoid or alleviate acute leptospirosis and other infectious diseases.

In our study, we did not dissect the mechanisms by which the macrophages exerted their therapeutic effects. However, we observed *ex vivo* that infected macrophages previously trained with CL429 had strikingly high NO production that resulted in killing of leptospires. These results strongly suggest that the enhanced NO response of peritoneal cells may participate in the reduction of leptospiral loads observed *in vivo* in CL429-treated mice. Whether enhanced levels of antimicrobial peptides and proteins involved in reactive oxygen species production are also triggered remains to be studied.

Although upregulation of PRRs has been shown upon training with BCG [38], we did not observe an upregulation of NOD1, NOD2 or TLR4 mRNA in BMMs. However, we used BMM since we were limited by the number of peritoneal cells and this result can be considered with caution. Indeed, upon training with CL429, peritoneal and bone marrow-derived macrophages did not behave exactly the same. Although secretion of IL6 was increased in peritoneal and bone marrow derived macrophages, the chemokines KC and RANTES showed opposite trends, with KC secretion increased in trained BMMs and not changed in peritoneal cells, whereas the opposite was true for RANTES.

Since trained immunity has been mostly studied in human monocytes/macrophages that do not produce NO, NO has not been yet routinely associated with TI. Here, we showed that NO production, known as a potent antileptospiral compound [18, 19], was an excellent read-out of TI, which may also explain how mice might control the infection. Interestingly, we observed that CL429 injection led to a systemic effect, as observed for the responsiveness of splenic NKs from treated mice, which produced more IFN-γ. NO production was enhanced in presence of IFN-γ, a cytokine produced by T and NK cells. We did not observe NO production in cells from Rag2-γc mice (lacking B, T and NK cells), and we showed in WT mice that NK cells, not T-cells, were the main producers of IFN-γ. Augmented cytokine production by trained macrophages could stimulate NK cells to secrete more IFN-γ, or NK cells themselves could be “trained”, as previously reported [28]. Interestingly, a protective role of IFN-γ against renal colonization with pathogenic leptospires has recently been suggested [45]. Considering that our present study shows reduced kidney load at day 15 post-infection, it would be interesting, as was proposed by Zuerner in 2011 [46], to further prime the NK cells to limit renal colonization, which we still observed after CL429 treatment at one month post-infection. Whether NK could be trained using TLR and NOD agonists other than CL429 to limit the renal colonization after infection with pathogenic leptospires will be the focus of future studies.

Consistent with a trained immunity effect that provides non-specific protection, peritoneal and bone marrow-derived macrophages from CL429-treated mice responded better than those from nontreated mice to *ex vivo* stimulation with LPS from *E*. *coli* and with different serovars of pathogenic leptospires isolated worldwide (Icterohaemorragiae strain Verdun, a French isolate, Copenhageni strain Fiocruz, a Brazilian strain and Manilae strain L495, isolated in Philippines). Of note, the 3 different strains stimulated trained macrophages and human monocytes to the same extent. Efficient vaccines against leptospirosis are notoriously difficult to establish because of the numerous leptospiral serovars identified on the basis of the highly immunogenic but variable O-antigen portions of the lipopolysaccharide. Therefore, only serovar-specific vaccines are available. Interestingly, our study suggests that CL429 may be used as a prophylactic treatment to alleviate the severity of leptospirosis, independently of the *Leptospira* serovar.

*In vitro* studies have shown that NOD2 is associated with innate immune memory effects [22, 38]. Conversely, TLR2 has been reported to induce tolerance [22], the opposite functional effect of innate immune memory, in which the immune response to second encounters is significantly reduced. Our study showed that CL429, a bi-functional TLR2/NOD2 agonist, triggered innate immune memory in a mouse model but also, in a certain low concentration range, boosted human monocyte responses. Their functional response led to the production of more IL-6 upon stimulation with several leptospiral pathogenic strains. Interestingly, at comparable molar concentrations, the CL429 effect was slightly higher than MDP, which was used as a positive control for training [38]. This synergistic effect of CL429 has been previously highlighted by Pavot *et al*., [36] who first tested the effect of CL429 on the activation of dendritic cells, and by Rice *et al*., [35], who showed in mice that CL429 was better than NOD2 or TLR2 agonists alone to alleviate a secondary pulmonary viral infection. Here we further demonstrated that the immunomodulatory role of CL429 indeed corresponded to trained immunity, as previously suggested [35]. Of note, *Borrelia burgdorferi*, another spirochete, has been used as a non specific secondary stimulant of ß-glucan-trained human monocytes [47], which suggests that trained immunity could also be used to limit Lyme disease.

We have recently shown that pathogenic *Leptospira* escape the NOD1 and NOD2 response [12]. NOD2 has been involved in adaptive immunity and shown to instruct the onset of antigen-specific T and B cell immunity *in vivo* in mice. Moreover, NOD2 stimulation shifts the immune response towards a Th2-type profile characterized by the induction of IL-4 and IL-5 by T cells and by IgG1 antibody responses [48]. Th2 confers immunity to extracellular pathogens. Even though *L*. *interrogans* have been described intracellularly in macrophages [15, 17, 41], *L*. *interrogans* are extracellular pathogens that replicate in blood or in the lumen of renal proximal tubules. Whether leptospiral escape from NOD1/2 signaling could consequently limit efficiency of macrophages to phagocytose leptospires and, thereby, alter the onset of adaptive immunity is under investigation. However, in this study, we showed that CL429, a NOD2 agonist, activated macrophage function. Vaccine grade CL429 is available and could be used as an adjuvant [36] combined with conserved antigens of pathogenic leptospires, potentially establishing novel vaccines against leptospirosis. Likewise for BCG, which is at the origin of the discovery of trained immunity and has been used as a platform for many vaccines, we may hypothesize that CL429 could increase the efficiency of leptospiral vaccines. Thus, a recombinant BCG-based leptospiral vaccine expressing LipL32 (the most abundant leptospiral lipoprotein) rescued half of the treated hamsters from lethal challenge (2x LD50) with *L*. *interrogans* 70 days post-immunization compared to the rescue of one-fifth with the control BCG [49].

A limitation of the present study is the route of CL429 administration and leptospire infection. We only studied the training effect of CL429 after intraperitoneal injection and leptospire challenge using the same route, neither of which are physiologic, nor are they adapted to vaccine trials. Other mouse models of mucosal [50] or subcutaneous infection of leptospires should be used to test the effect of CL429.

In conclusion, we showed that CL429 treatment in mice and in human cells triggered innate immune memory that helped the host to fight acute leptospiral infection, independently of the serovar of *L*. *interrogans*. Our study demonstrates for the first time that manipulation of macrophages and other innate immune cells through the induction of innate immune memory could have a positive effect *in vivo* in mice to alleviate acute leptospirosis. Furthermore, it enhanced *ex vivo* the inflammatory response of human monocytes towards leptospires. This result led us to hypothesize that CL429 might be used as a prophylactic treatment to alleviate human and animal leptospirosis. Thus, this work paves the way to the design of new prophylactic strategies against leptospirosis. Although a universal vaccine against leptospires is still a long-term goal [3], we envision the use TLR and/or NLR combined agonists, which are known to train immunity, as adjuvants for future leptospiral vaccines.

## Supporting information

S1 Fig General gating strategy and results after CL429 treatment. A) The gating strategy indicating the markers used to phenotype macrophages (Macro), neutrophils (Neutro), monocytes (Mono), NK-, B- and T-cells, for blood, peritoneal lavage and spleen analyses. (B-D) Cell populations determined by flow cytometry analysis 2 weeks after the first PBS or CL429 treatment of peritoneal cells (B), whole blood (C) and spleen (D). Plots corresponds to 1 experiment with *n* = 4 mice.
(TIF)

S2 Fig Controls. A) Viability controls of *Leptospira interrogans* in the presence of the drugs (liposomes and CL429) used in this study. Bioluminescent leptospires were used to assess the viability of bacteria upon incubation with PBS, clodronate-containing liposomes or the TLR2/NOD2 bifunctional agonist CL429; each were used at the concentration that was used for peritoneal injection. Only live leptospires will produce bioluminescence upon exogenous administration of the luciferase substrate luciferin. Bacterial suspensions (100 μL) treated as indicated were placed at different time-points in a white flat bottom 96-well plate. A volume of 100 μL luciferin was injected into the well, and bioluminescence was measured with an integration time of 10 seconds. Each curve was performed using 3 technical replicates. No difference was observed in the survival of leptospires at any of the tested time-points. B) *Lactobacillus plantarum* strain 256 triggers NOD2 and TLR2 signaling. HEK293T were grown in complete DMEM medium (high glucose, GlutaMAX supplement, pyruvate and 10% heat inactivated fetal calf serum) (Gibco) and seeded at 5x105 cells in a volume of 1 ml per well in 24 well-plates. The following day, cells were transiently transfected with human TLR2 or with human NOD2, as previously described. The cells were stimulated with CL429 (1 μM) and *L*. *plantarum* 256 (L.p) diluted in PBS from frozen stocks (MOI of 100, 10 and 1). After 2 h of stimulation, gentamicin (50 μg/ml) (Gibco) was added to avoid overgrowth of the bacteria. In the case of NOD2 transfection, the stimulation was performed 30 minutes before transfection to allow the muropeptides to gain access to the cells along with the transfection reagent. In the case of TLR2 transfection, the stimulation was performed for 6 h, 24 h after transfection. The cells were lysed 24 h after stimulation and luciferase activities measured with a CENTRO luminometer.
(TIF)

S3 Fig PRR expression profiles in bone marrow derived macrophages (BMMs) from PBS- and CL429-treated mice. BMMs (106 cells /mL) from *n* = 6 CL429-treated and *n* = 6 PBS-treated mice were individually seeded in 24 well-plates and rested for 2 days before stimulation for 24 h with live *Leptospira interrogans* serovar Manilae at MOI of 100 or not stimulated (NS). RNA purification, reverse transcription into cDNA and real-time RT-PCR using mouse HPRT, NOD1, NOD2 and TLR4 primers and FAM TAMRA probes were performed as described previously (Tourneur et al., PLoS Pathogens, 2013). RT-PCR reactions were run on a Step one Plus real-time PCR apparatus using the ΔΔCt program (Applied Biosystems) according to the manufacturer’s instructions. Data are expressed as the fold change in gene expression of mouse NOD1, NOD2 and TLR4 relative to HPRT compared to the non-stimulated, PBS-treated BMMs.
(TIF)

S4 Fig Peritoneal macrophages are still trained at 8 weeks post-CL429 treatment. A) Cell populations in peritoneal lavage, 8 weeks after PBS or CL429 treatment of mice were determined by flow cytometry analysis according to the gating indicated in S1A Fig. Plots correspond to 1 experiment with *n* = 3 PBS-treated and *n* = 4 CL429-treated mice. B) Proportion of macrophages (CD11b^+^/F480^+^) in each well 20 h post-stimulation. C) Representative histograms for iNOS expression in peritoneal CD11b^+^/F4/80^+^ (macrophages) cells from PBS-treated and CL429-treated mice 24 h after *ex vivo* stimulation with LPS or leptospires compared to non stimulated (NS). Supernatants were removed, and cells were detached from the plastic after 30 minutes with 10 mM EDTA. EDTA was then removed, and the cells were washed and resuspended in PBS 1% FCS 2 mM EDTA for surface staining. For intracellular staining, the cells were incubated with antibody in 100 μL InsidePerm (Miltenyi Biotec) overnight at 4°C. The cells were washed and resuspended in PBS 1% FCS 2 mM EDTA and processed as indicated in the Materials and Methods section. PBS and CL429 indicate mouse treatments performed 8 weeks prior. Representative histograms for iNOS expression in (CD11b^+^/F4/80^+^) macrophages. The dashed and solid lines indicate non stimulated and stimulated cells, respectively. D) The plot corresponds to the quantitative (mean fluorescence intensity) analysis of the iNOS protein present in macrophages stimulated for 24 h with LPS and 3 different leptospiral serovars. Data are from one experiment with cells from *n* = 3 CL429-treated mice and *n* = 2 PBS-treated mice.
(TIF)

S5 Fig Long-term systemic effect of CL429 treatment in spleen. (A-D) Flow cytometry analysis of splenic cells from PBS-treated and CL429-treated mice 24 h post-stimulation with LPS and different leptospires compared to non stimulated (NS). Supernatants were removed, and cells were resuspended in PBS 1% FCS 2 mM EDTA for surface staining using the antibodies indicated in Table S1. For intracellular staining, the cells were incubated with antibody in 100 μL of InsidePerm (Miltenyi Biotec) overnight. The cells were washed, resuspended in PBS 1% FCS 2 mM EDTA and processed as indicated in the Material and methods section. PBS and CL429 indicate the mouse treatments performed 3 months prior. A) Cell populations in whole spleen 2 months after PBS or CL429 treatment of mice as determined by flow cytometry analysis and according to the gating indicated in S1A Fig. Plots correspond to 1 experiment with *n* = 3 PBS-treated and *n* = 4 CL429-treated mice. B) Quantitative data for the percentage of NK cells (NK1.1^+^/CD3^-^) among splenic cells from mice treated 3 months prior with CL429 or PBS in plated cells 24 h post-stimulation. Data are representative of 1 experiment with *n* = 6 female mice for each treatment. C) Representative histograms of flow cytometry analysis of IFN-γ produced by NK (NK1.1^+^/CD3^-^) cells from spleens of mice treated 3 months prior with CL429 or PBS, 24 h post-stimulation with LPS or live leptospires. The dashed and solid lines indicate non stimulated and stimulated cells, respectively. Data are representative of 1 experiment with *n* = 5 female mice. D) Quantitative data for IFN-γ produced by NK cells from spleens of mice treated 3 months prior with CL429 or PBS 24 h post-stimulation with LPS or live leptospires, Data are representative of one experiment with *n* = 5 female mice.
(TIF)

S6 Fig Long-term systemic effect of CL429 treatment in BMMs. A) Pro-inflammatory cytokine (IL-6) and chemokine (KC and RANTES) profiles determined by ELISA in the supernatants of BMMs cells from mice treated 3 months prior with CL429 or PBS, 24 h post-stimulation with LPS or live leptospires. Data are representative of 1 experiment (*n* = 5 mice per group, treated individually). B) Nitric oxide (NO) production by BMMs at 24 h after stimulation, as assessed by the Griess reaction. Data correspond to 1 experiment (*n* = 5 mice per group, treated individually). Statistical analysis was performed by 2-way ANOVA comparing PBS vs CL429 for each stimulation. * *p*-value < 0.05; ** *p*-value < 0.01; *** *p*-value < 0.001.
(TIF)

S7 Fig Human monocyte viability at 24 h post-stimulation. A) Chronogram of the *in vitro* training of human monocytes isolated from healthy donor blood. B) Control of cell viability by MTT. The MTT assay was performed 24 h post-stimulation, 5 days after the training. For the first stimulation (training), cells were stimulated for 24 h with PBS as a control, MDP (0.1 μM) or CL429 (0.1 μM). The cells were then stimulated a second time 5 days later with PBS, LPS (100 ng/mL) or live L. interrogans serovars Manilae and Fiocruz at MOI of 100. After stimulation, the supernatants were removed and the cells incubated with 0.25 mg/mL MTT (Sigma-Aldrich) for 2 h in RPMI. MTT crystals formed in viable cells were dissolved with HCl:isopropanol (V/V) for 30 minutes, and the absorbance at 595 nm was recorded. Each graph corresponds to the indicated second stimulation for all tested donors. Data represent the mean and standard deviation of the absorbance values of 4 technical replicates.
(TIF)
