# Supplementary figures and images for: Innate immune memory through TLR2 and NOD2 contributes to the control of Leptospira interrogans infection
Source: PLoS Pathog. 2019 May 20;15(5):e1007811. doi: 10.1371/journal.ppat.1007811 (PMC6544334; doi:10.1371/journal.ppat.1007811)

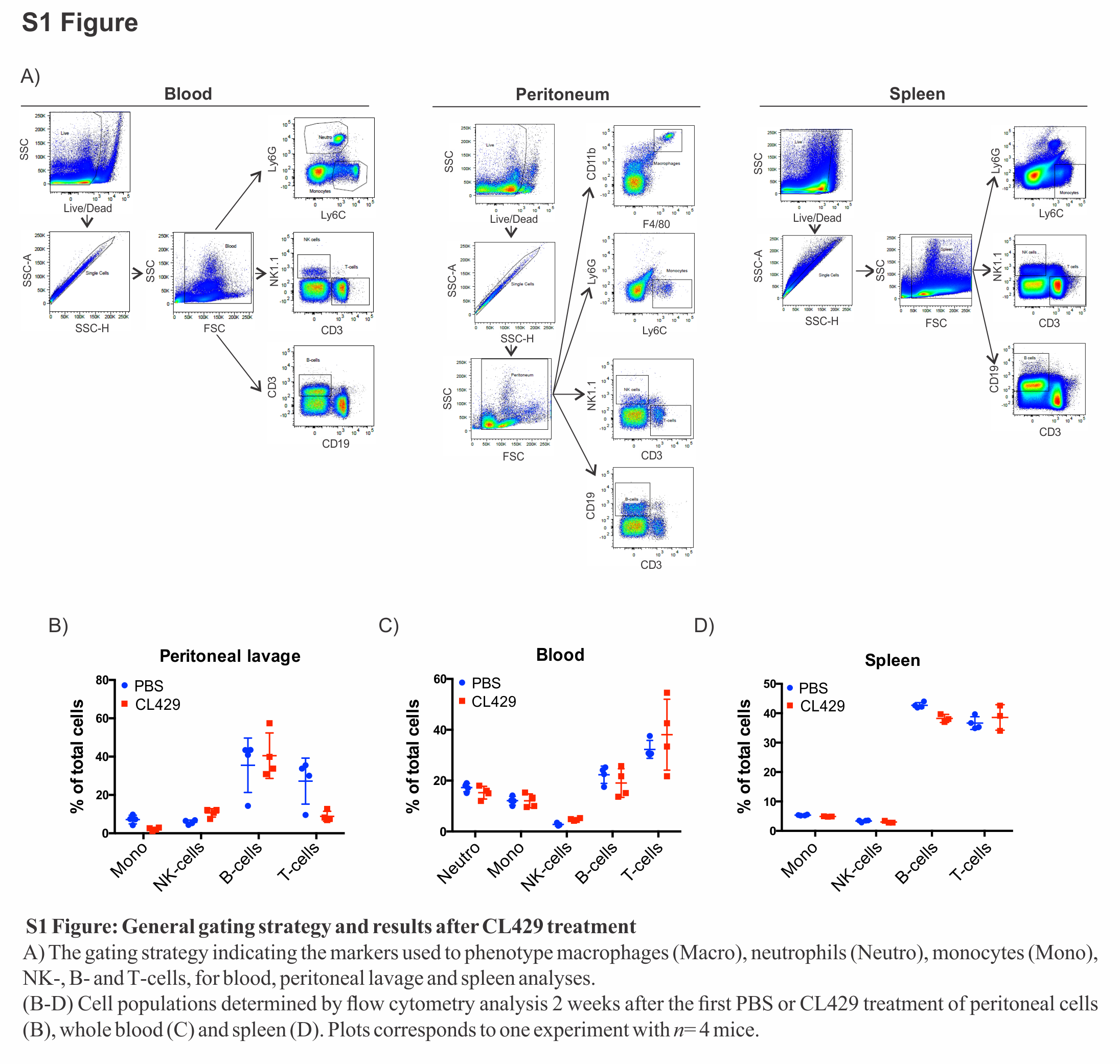

Supplement: S1 Fig — A) The gating strategy indicating the markers used to phenotype macrophages (Macro), neutrophils (Neutro), monocytes (Mono), NK-, B- and T-cells, for blood, peritoneal lavage and spleen analyses. (B-D) Cell populations determined by flow cytometry analysis 2 weeks after the first PBS or CL429 treatment of peritoneal cells (B), whole blood (C) and spleen (D). Plots corresponds to 1 experiment with n = 4 mice. (TIF) [file ppat.1007811.s001.tif]

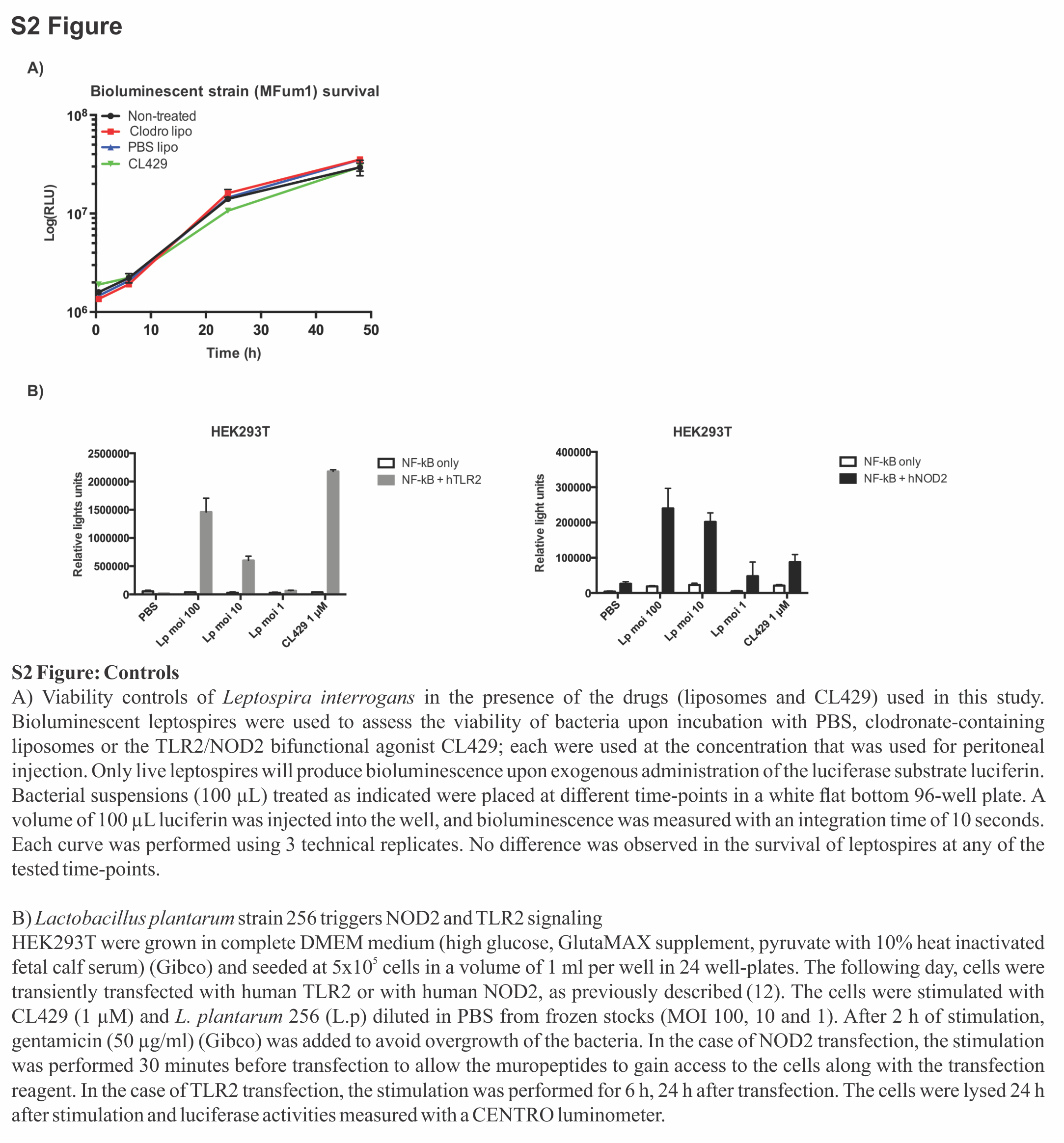

Supplement: S2 Fig — A) Viability controls of Leptospira interrogans in the presence of the drugs (liposomes and CL429) used in this study. Bioluminescent leptospires were used to assess the viability of bacteria upon incubation with PBS, clodronate-containing liposomes or the TLR2/NOD2 bifunctional agonist CL429; each were used at the concentration that was used for peritoneal injection. Only live leptospires will produce bioluminescence upon exogenous administration of the luciferase substrate luciferin. Bacterial suspensions (100 μL) treated as indicated were placed at different time-points in a white flat bottom 96-well plate. A volume of 100 μL luciferin was injected into the well, and bioluminescence was measured with an integration time of 10 seconds. Each curve was performed using 3 technical replicates. No difference was observed in the survival of leptospires at any of the tested time-points. B) Lactobacillus plantarum strain 256 triggers NOD2 and TLR2 signaling. HEK293T were grown in complete DMEM medium (high glucose, GlutaMAX supplement, pyruvate and 10% heat inactivated fetal calf serum) (Gibco) and seeded at 5x10^5^ cells in a volume of 1 ml per well in 24 well-plates. The following day, cells were transiently transfected with human TLR2 or with human NOD2, as previously described. The cells were stimulated with CL429 (1 μM) and L. plantarum 256 (L.p) diluted in PBS from frozen stocks (MOI of 100, 10 and 1). After 2 h of stimulation, gentamicin (50 μg/ml) (Gibco) was added to avoid overgrowth of the bacteria. In the case of NOD2 transfection, the stimulation was performed 30 minutes before transfection to allow the muropeptides to gain access to the cells along with the transfection reagent. In the case of TLR2 transfection, the stimulation was performed for 6 h, 24 h after transfection. The cells were lysed 24 h after stimulation and luciferase activities measured with a CENTRO luminometer. (TIF) [file ppat.1007811.s002.tif]

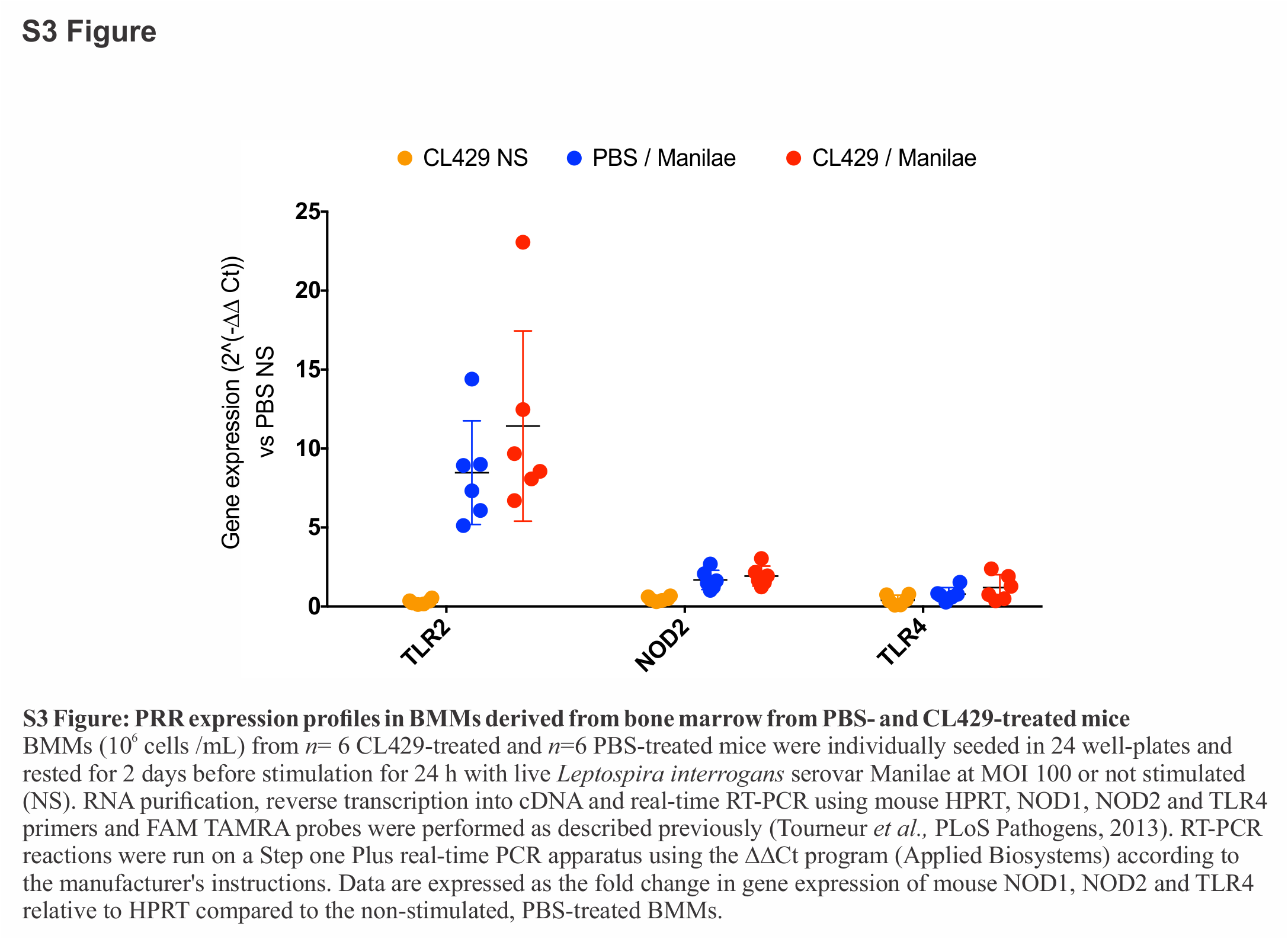

Supplement: S3 Fig — BMMs (106 cells /mL) from n = 6 CL429-treated and n = 6 PBS-treated mice were individually seeded in 24 well-plates and rested for 2 days before stimulation for 24 h with live Leptospira interrogans serovar Manilae at MOI of 100 or not stimulated (NS). RNA purification, reverse transcription into cDNA and real-time RT-PCR using mouse HPRT, NOD1, NOD2 and TLR4 primers and FAM TAMRA probes were performed as described previously (Tourneur et al., PLoS Pathogens, 2013). RT-PCR reactions were run on a Step one Plus real-time PCR apparatus using the ΔΔCt program (Applied Biosystems) according to the manufacturer’s instructions. Data are expressed as the fold change in gene expression of mouse NOD1, NOD2 and TLR4 relative to HPRT compared to the non-stimulated, PBS-treated BMMs. (TIF) [file ppat.1007811.s003.tif]

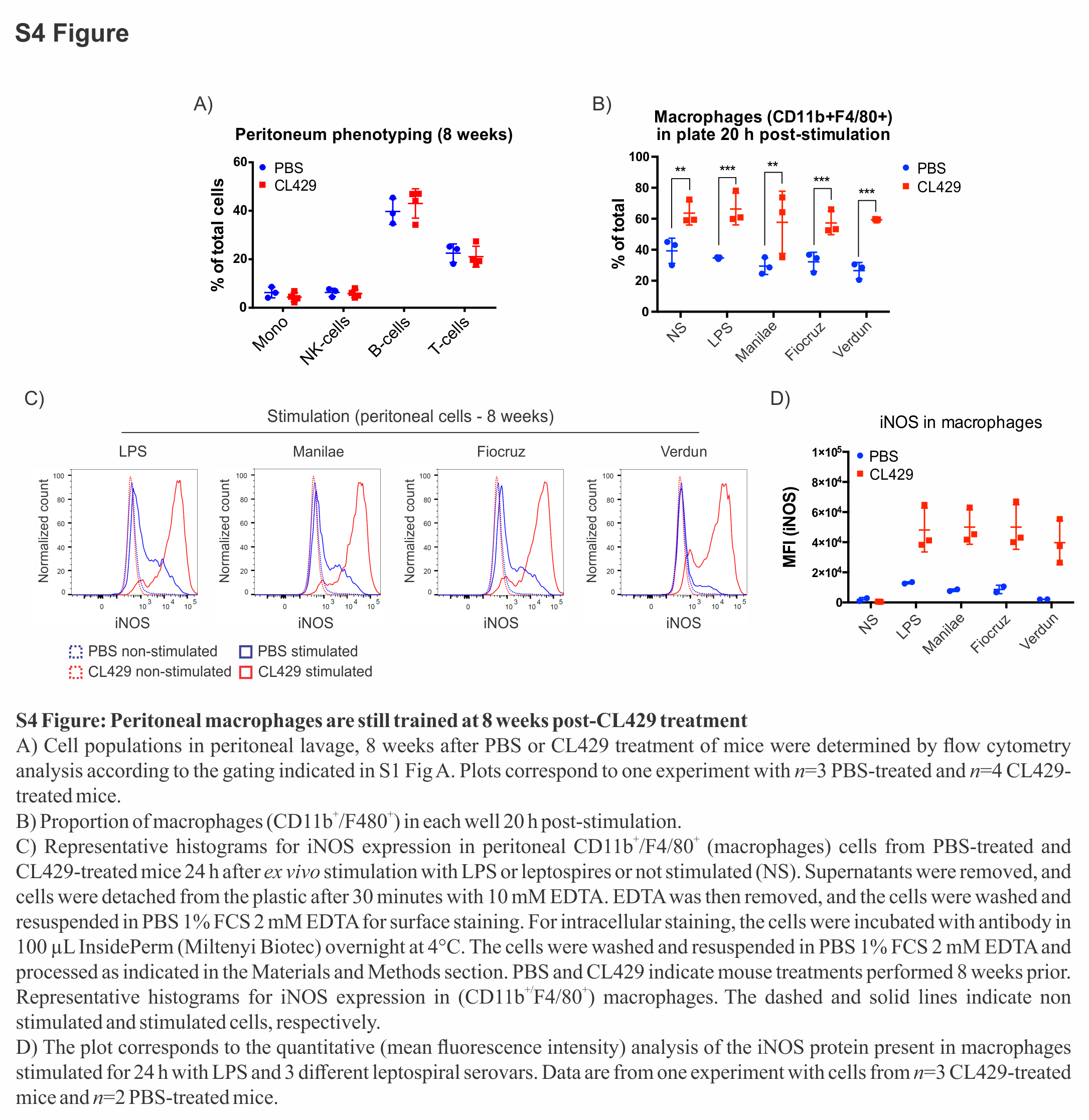

Supplement: S4 Fig — A) Cell populations in peritoneal lavage, 8 weeks after PBS or CL429 treatment of mice were determined by flow cytometry analysis according to the gating indicated in S1A Fig. Plots correspond to 1 experiment with n = 3 PBS-treated and n = 4 CL429-treated mice. B) Proportion of macrophages (CD11b+/F480+) in each well 20 h post-stimulation. C) Representative histograms for iNOS expression in peritoneal CD11b+/F4/80+ (macrophages) cells from PBS-treated and CL429-treated mice 24 h after ex vivo stimulation with LPS or leptospires compared to non stimulated (NS). Supernatants were removed, and cells were detached from the plastic after 30 minutes with 10 mM EDTA. EDTA was then removed, and the cells were washed and resuspended in PBS 1% FCS 2 mM EDTA for surface staining. For intracellular staining, the cells were incubated with antibody in 100 μL InsidePerm (Miltenyi Biotec) overnight at 4°C. The cells were washed and resuspended in PBS 1% FCS 2 mM EDTA and processed as indicated in the Materials and Methods section. PBS and CL429 indicate mouse treatments performed 8 weeks prior. Representative histograms for iNOS expression in (CD11b+/F4/80+) macrophages. The dashed and solid lines indicate non stimulated and stimulated cells, respectively. D) The plot corresponds to the quantitative (mean fluorescence intensity) analysis of the iNOS protein present in macrophages stimulated for 24 h with LPS and 3 different leptospiral serovars. Data are from one experiment with cells from n = 3 CL429-treated mice and n = 2 PBS-treated mice. (TIF) [file ppat.1007811.s004.tif]

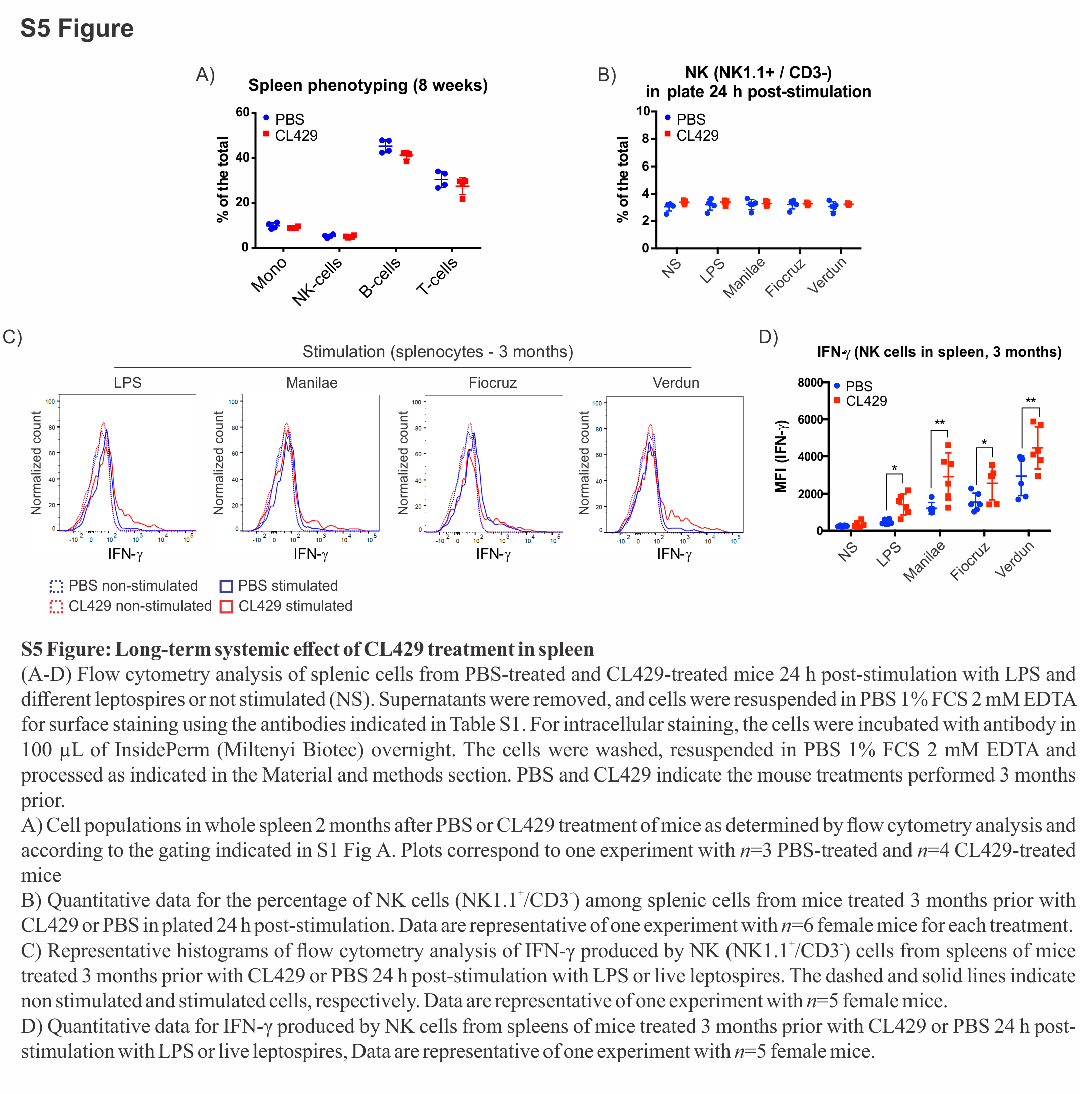

Supplement: S5 Fig — (A-D) Flow cytometry analysis of splenic cells from PBS-treated and CL429-treated mice 24 h post-stimulation with LPS and different leptospires compared to non stimulated (NS). Supernatants were removed, and cells were resuspended in PBS 1% FCS 2 mM EDTA for surface staining using the antibodies indicated in Table S1. For intracellular staining, the cells were incubated with antibody in 100 μL of InsidePerm (Miltenyi Biotec) overnight. The cells were washed, resuspended in PBS 1% FCS 2 mM EDTA and processed as indicated in the Material and methods section. PBS and CL429 indicate the mouse treatments performed 3 months prior. A) Cell populations in whole spleen 2 months after PBS or CL429 treatment of mice as determined by flow cytometry analysis and according to the gating indicated in S1A Fig. Plots correspond to 1 experiment with n = 3 PBS-treated and n = 4 CL429-treated mice. B) Quantitative data for the percentage of NK cells (NK1.1+/CD3-) among splenic cells from mice treated 3 months prior with CL429 or PBS in plated cells 24 h post-stimulation. Data are representative of 1 experiment with n = 6 female mice for each treatment. C) Representative histograms of flow cytometry analysis of IFN-γ produced by NK (NK1.1+/CD3-) cells from spleens of mice treated 3 months prior with CL429 or PBS, 24 h post-stimulation with LPS or live leptospires. The dashed and solid lines indicate non stimulated and stimulated cells, respectively. Data are representative of 1 experiment with n = 5 female mice. D) Quantitative data for IFN-γ produced by NK cells from spleens of mice treated 3 months prior with CL429 or PBS 24 h post-stimulation with LPS or live leptospires, Data are representative of one experiment with n = 5 female mice. (TIF) [file ppat.1007811.s005.tif]

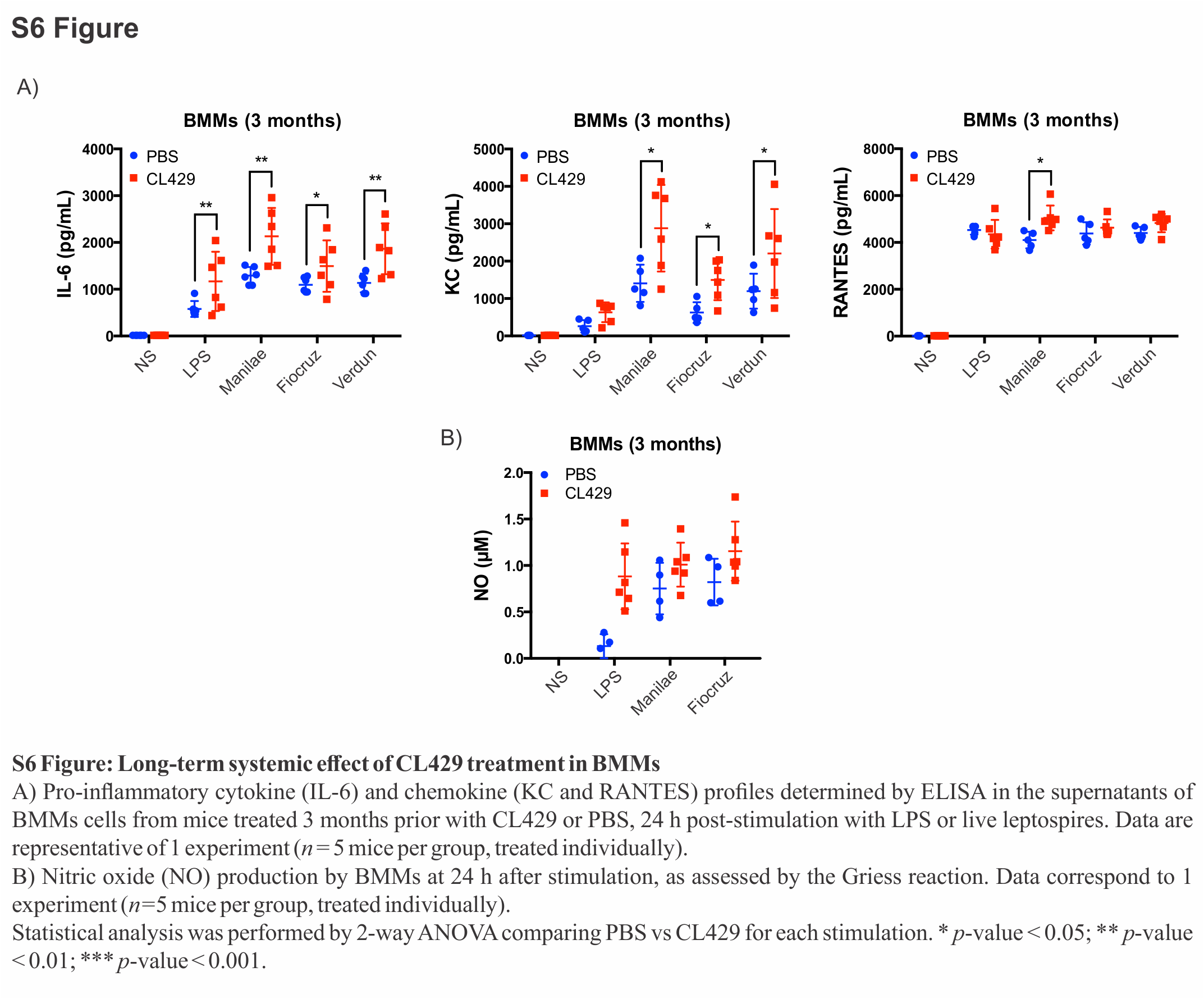

Supplement: S6 Fig — A) Pro-inflammatory cytokine (IL-6) and chemokine (KC and RANTES) profiles determined by ELISA in the supernatants of BMMs cells from mice treated 3 months prior with CL429 or PBS, 24 h post-stimulation with LPS or live leptospires. Data are representative of 1 experiment (n = 5 mice per group, treated individually). B) Nitric oxide (NO) production by BMMs at 24 h after stimulation, as assessed by the Griess reaction. Data correspond to 1 experiment (n = 5 mice per group, treated individually). Statistical analysis was performed by 2-way ANOVA comparing PBS vs CL429 for each stimulation. * p-value < 0.05; ** p-value < 0.01; *** p-value < 0.001. (TIF) [file ppat.1007811.s006.tif]

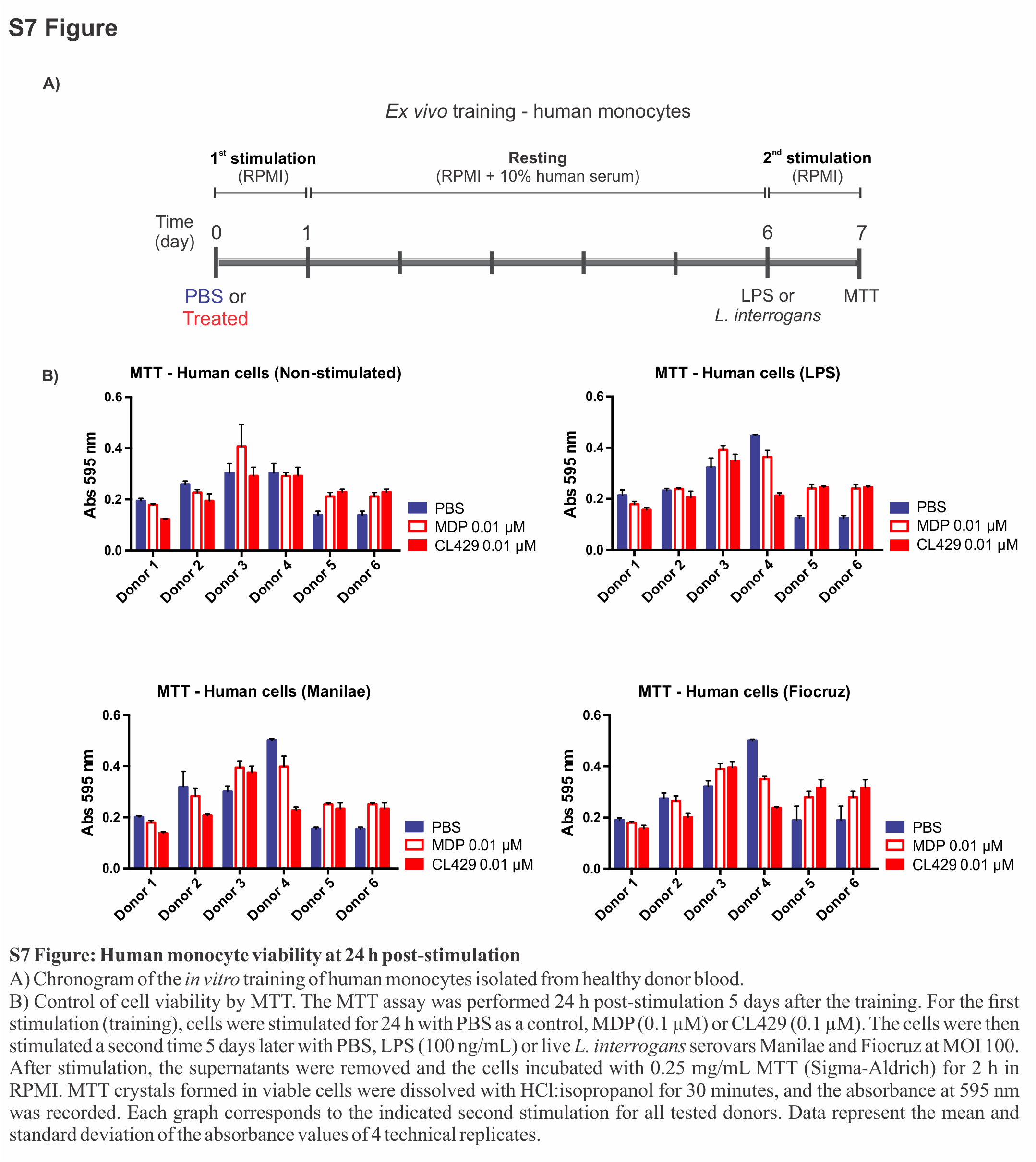

Supplement: S7 Fig — A) Chronogram of the in vitro training of human monocytes isolated from healthy donor blood. B) Control of cell viability by MTT. The MTT assay was performed 24 h post-stimulation, 5 days after the training. For the first stimulation (training), cells were stimulated for 24 h with PBS as a control, MDP (0.1 μM) or CL429 (0.1 μM). The cells were then stimulated a second time 5 days later with PBS, LPS (100 ng/mL) or live L. interrogans serovars Manilae and Fiocruz at MOI of 100. After stimulation, the supernatants were removed and the cells incubated with 0.25 mg/mL MTT (Sigma-Aldrich) for 2 h in RPMI. MTT crystals formed in viable cells were dissolved with HCl:isopropanol (V/V) for 30 minutes, and the absorbance at 595 nm was recorded. Each graph corresponds to the indicated second stimulation for all tested donors. Data represent the mean and standard deviation of the absorbance values of 4 technical replicates. (TIF) [file ppat.1007811.s007.tif]
